# The efficacy of a brief intervention to reduce alcohol use in persons with HIV in South Africa, a randomized clinical trial

**DOI:** 10.1371/journal.pone.0220799

**Published:** 2019-08-20

**Authors:** Diana Huis in ‘t Veld, Chellafe Ensoy-Musoro, Supa Pengpid, Karl Peltzer, Robert Colebunders

**Affiliations:** 1 Department of Internal Medicine and Infectious Diseases, UZ Ghent, Ghent, Belgium; 2 Department of Epidemiology and Social Medicine, University of Antwerp, Antwerp, Belgium; 3 FWO Research Foundation Flanders, Brussels, Belgium; 4 Interuniversity Institute for Biostatistics and Statistical Bioinformatics, University of Hasselt, Diepenbeek, Belgium; 5 ASEAN Institute for Health Development, Mahidol University, Nakhonpathom, Thailand; 6 Department of Research and Innovation, University of Limpopo, Sovenga, South Africa; 7 Global Health Institute, University of Antwerp, Antwerp, Belgium; Azienda Ospedaliera Universitaria di Perugia, ITALY

## Abstract

**Background:**

Since there is a high prevalence of high risk alcohol use in patients with HIV in Africa, with negative health effects, there is a need for short interventions to reduce alcohol use.

**Methods:**

We studied the efficacy of a short intervention aiming to reduce alcohol use based on the Information-Motivation-Behavioural Skills Model in patients with HIV with high alcohol use (measured by AUDIT). The study was performed in three outpatient clinics in South Africa. The intervention group received in one-session intervention a personalized feedback on AUDIT results trying to make people aware that they are in the medium- or high-risk drinking category. Both the intervention and the control group received a health education leaflet.

**Results:**

A total of 560 patients participated in the study with a follow up of 1 year. There was a significant decrease in total AUDIT scores between baseline and follow up points 1 (5 months) and 2 (1 year) in both groups. There was no significant decrease between time points 1 and 2. However, between the intervention and control groups there was no difference in reduction of alcohol use to abstinence or low risk alcohol use over time as there was no difference in absolute decrease in AUDIT-score or percentage of change in AUDIT score. The intervention had no influence on the quality of life outcomes, depression scores, stigma, tobacco use, viral load and therapy adherence at both time points. In all secondary outcomes, there was no significant interaction between intervention and time.

**Conclusion:**

The brief intervention was not successful at reducing alcohol use both 5 and 12 months after the intervention. However, there was a beneficial effect on reported hazardous or harmful alcohol use at least over a short term follow up period in both study groups. It might be that only an interview and/or the distribution of a health leaflet can be successful in reducing alcohol use but this needs to be investigated with more objective measures of alcohol use. To sustain an effect, most likely repetitive contacts with hazardous or harmful alcohol drinkers will be needed during a long follow up period.

## Introduction

The prevalence of alcohol use disorders among patients with HIV is high in sub Saharan Africa. Previously we showed that of patients with HIV visiting primary health care clinics in South Africa up to 25.1% of the total group, and up to 40.3% of male patients were hazardous drinkers (defined as a quantity or pattern of alcohol consumption placing patients at risk for adverse health events) or harmful drinkers (defined as alcohol consumption resulting in adverse events) [1]. Since the prevalence of HIV in the adult population in South Africa is estimated to be 18.8% in 2017 [2], a considerable amount of people is exposed to additional health risks by consuming alcohol.

Several studies showed the negative associations of alcohol use in patients with HIV. The prevalence of HIV infection is higher in alcohol users [3–6], most likely due to the high-risk sexual behaviour associated with alcohol use [7–10]. There is a late presentation to care and return visits to the clinic are missed more often [11]. Alcohol use is related to lower adherence levels for ART [12,13] and to various (negative) patterns of ART taking [14]. There is a high prevalence of liver fibrosis in patients with HIV and alcohol use [15]. Moreover, alcohol use is a predictor of delay in diagnosis and treatment of tuberculosis in patients with HIV [16].

Therefore, there is a need to identify patients with HIV with an unhealthy alcohol use to test interventions aimed at reducing alcohol use. Previously conducted studies in patients with HIV in sub-Saharan Africa mostly focus on lowering sexual risk behaviour by reducing alcohol intake before sexual contacts [17].

Given the lack of human and financial resources in sub-Africa there is a need to evaluate whether a brief and simple intervention is able to identify a real or potential alcohol problem and to motivate an individual to do something about it. Brief interventions have been used for a long time to change behaviour. By definition they are brief, usually conducted in a one-to-one situation and involve raising awareness of the problem, share knowledge and get the person involved to think about making changes to his or her behaviour. It can be delivered in one or multiple sessions. The design of brief interventions can differ in different settings. We conducted a randomized controlled trial to evaluate a brief intervention to reduce alcohol use based on the Information-Motivation-Behavioural Skills Model. This model has proved to be successful in different settings to change behaviour [18,19].

A systematic review and meta-analysis of brief interventions (different interventions were compared, ranging from Motivational Interviewing, cognitive-behavioural techniques or other methods) for alcohol reduction in primary care patients including 19 trials (9 in North America, 7 in Europe, 2 in Africa and 1 in Australia) with 5639 individuals showed that of 17 trials reporting a quantified outcome measure in alcohol consumption, 8 reported that brief intervention for alcohol reduction in primary care patients was effective in reducing alcohol consumption at 6 and 12 months [20]. Seven trials reported no significant effects. None of the studies reported negative effects [20]. One study performed in New York showed improvement in self-reported ART adherence and biological markers (CD4 cell count and HIV viral load) after an eight sessions behavioural intervention after 3 months. However, this result was not sustained at 6 months [21]. A multi component (including behavioural) intervention showed no significant differences in adherence, CD4 count, viral load or alcohol consumption [22]. A recent randomized trial testing the efficacy of a single, brief alcohol reduction intervention in men and women with HIV in Uganda by Wandera et al. showed a non-differential reduction in alcohol consumption in both intervention and control arms [23]. So results from different brief interventions studies using different intervention methods to reduce alcohol use in patients with HIV show inconsistent outcomes.

At the start of our trial there was no reported study on a single-session brief intervention aiming to reduce alcohol use in patients with HIV in Africa. The aim of our study was to evaluate the effectivity of a brief intervention to reduce alcohol use in patients living with HIV with hazardous and harmful drinking patterns attending a primary care setting in South Africa. Taking account of the overcrowded, overwhelmed HIV clinics in this setting, the goal was to develop a short, single-session, easy-to-use intervention to reduce alcohol use among patients with HIV. We hypothesized that patients in the intervention group would reduce their drinking levels more than patients in the control group.

## Methods

A randomized controlled clinical trial was performed in 3 HIV clinics based within primary health care clinics in townships surrounding Pretoria in South Africa comparing a brief intervention to reduce alcohol use with standard of care combined with distributing a health education leaflet on responsible drinking. In these clinics patients with HIV receive basic care and ART free of charge. The trial protocol was described before [24].

Patients with HIV-1 infection who were 18 years or older and who visited the selected outpatient clinics for their HIV care were eligible for this study. Patients with mental impairment, not able to provide informed consent; (assessed by the clinic health care workers), or those already undergoing alcohol reduction treatment (e.g. receiving specialised care in a private clinic) and pregnant women (since they needed to be referred to a alcohol reduction treatment program without delay) were excluded from this study. All consecutive outpatients who gave informed consent were screened for alcohol use. Details of this population have been published before [1]. If fulfilling the criteria for hazardous or harmful alcohol use according to the AUDIT at baseline, patients were randomized into an intervention or control group (1:1 ratio). Randomization was done by the trial coordinator (not involved in inclusion of patients) by concealed, centrally allocated computer-generated random numbers and made available for the research assistants in envelopes. Patients were followed-up at 3 (time-point 1) and 12 (time-point 2) months, which were the standard visit intervals for follow up of persons in the HIV care. In the event of a drop-out, at least 6 individual attempts were made to contact patients by telephone. Patients were offered a drink (soda or juice) during the interviews and received vouchers for supermarkets worth 100 South African Rand ($8.78).

### Data collection instruments

Research assistants administered a range of questionnaires at baseline, 3 months and 12 months follow up in English or Tswana, the most spoken local languages.

#### Socio-demographic characteristics

A researcher designed questionnaire was used to collect data on patients’ age, gender, population group, language, educational level, marital status, highest educational qualification, main source of family income and residential status.

#### Health related information

A researcher designed questionnaire and data extraction sheet were used to collect data on first positive HIV test, start date for ART, ART regimen, co-infections, co-medication, CD4 cell count at initiation of therapy and the latest measured value and HIV viral load.

#### Alcohol consumption

The 10-item Alcohol Use Disorder Identification Test (AUDIT) was used to assess alcohol use levels [25]. A score of 0 indicates alcohol abstinence. Scores of 1–6 for women and 1–7 for men indicate low risk alcohol use. AUDIT scores between 7 to 15 for women and 8 to 15 for men indicate hazardous drinking, which is defined as a quantity or pattern of alcohol consumption that places patients at risk for adverse health events, scores from 16–19 harmful drinking, which is defined as alcohol consumption that results in adverse events (for example physical or psychological harm) [26]. Scores of 20 and above indicate (possible) alcohol dependence. Cronbach alpha scores were 0.88 (for the whole screened group of 2249 patients), 0.46 at baseline, 0.81 and 0.78 for time points 1 and 2 respectively.

To reduce the stigma of alcohol use, the WHO suggests integrating the screening of alcohol use with screening for other health-related behaviours. Therefore, we included 2 questions on the use of tobacco products and anthropometric measurements were taken to assess the risk factor of overweight (height, weight and waist circumference).

#### Health related quality of life

The WHOQoL-HIVBREF questionnaire was used to evaluate the quality of life, which comprises the physical (domain 1), psychological (domain 2), level of independence (domain 3), social relationships (domain 4), environment (domain 5) and spirituality/religion/personal beliefs (domain 6) domains [27]. Two questions measure the overall quality-of-life directly (quality of life and health satisfaction). The higher the domain scores, the higher the perceived quality of life [28]. Cronbach alpha values for each of the six domain scores ranged from 0.72 to 0.85, showing acceptable internal consistency reliability [29].

#### Depression

The 10-item version of the Center for Epidemiologic Studies Depression (CES-D) Scale was used to assess symptoms of depression [30,31]. A cut-off score of ten or higher indicates the presence of significant depressive symptoms. The CES-D-10 is a valid, reliable screening tool for depression in South African populations [32].

#### Internalized AIDS stigma

The seven items internalized AIDS-related stigma scale for people infected with HIV was used to reflect self-defacing beliefs and negative perceptions of people living with HIV/AIDS [33].

#### Adherence to antiretroviral therapy

Adherence to ART was assessed only in patients on ART by self-reported adherence by recall of missed doses during the previous 7 days [34]. The second method used to measure adherence was the visual analogue scale which provided an overall adherence assessment for a 1-month period. A score of 100% is considered as full adherence, a score between ≥95% and <100% as partial adherence, and below 95% as non-adherence.

### Intervention

Research assistants (four nurses), not attached to the clinics, were trained to deliver the interventions to the patients and follow them up. The training consisted of (video recorded) role play, general skills training techniques and training on alcohol-, HIV- and sexual-related issues. The sites were visited twice weekly by one of the investigators to observe whether there was adherence to the protocol and to offer support and supervision to the research assistants. During regular study team meetings occurring problems were discussed and solved.

#### Intervention group

The brief intervention was based on the WHO brief intervention package for hazardous and harmful drinking, which is based on the Information-Motivation-Behavioural Skills Model [35]. Patients in the intervention group received personalized feedback on the AUDIT results, informing them that screening results indicated they belong to a hazardous or harmful alcohol use category. Specific harm(s) were itemized and seriousness of the situation was emphasized, trying to make people aware that they are in the medium- or high-risk drinking category. A health education leaflet on responsible drinking, emphasizing the idea of sensible limits, was given and discussed. Subsequently advice and brief counselling on reducing excessive drinking was offered during a one-session intervention including working through the first three sections of the problem-solving manual (describing what low-risk drinking constitutes, how to change drinking habits and good reasons for drinking less) while mentioning the value of reviewing the other sections (high-risk situations, what to do when tempted or bored, how to stick to the plan, involving a helper and creating a habit-breaking plan); and mention the 3-month and 12-month follow-up.

#### Control group

Patients in the control group only received a health education leaflet on responsible drinking.

### Statistical analysis

The primary outcome of the study was reduction from hazardous (AUDIT between 7 to 15 for women and 8 to 15 for men) or harmful (AUDIT between 16 to 19) alcohol use to alcohol abstinence (AUDIT score 0) or low risk alcohol use (AUDIT score 1–6 for women and 1–7 for men) at time points 1 and 2.

Secondary outcomes were reduction in absolute numbers and reduction in percentages in AUDIT score at time points 1 and 2. To measure any other possible effects of the intervention, other secondary outcomes were changes in medically related outcomes (last measured CD4 cell count, last measured HIV viral load and ART adherence), quality of life outcomes (measured with the WHOQoL-HIVBREF tool), depression score, level of experienced stigma and tobacco use.

The sample size was calculated using Open Epi (version 3) [36]. Based on the current mean AUDIT score of 12 among patients with HIV, it was assumed that the intervention will reduce the current AUDIT score by 12% to 10.6 [37,38]. Based on this assumption, the estimated sample size would allow us with 80% power (5% level of significance) to detect the difference of 12% between the two groups. This would give a minimum of 99 patients per arm. It was expected that 20% of participants may be lost prior to completing the 3-month and 12-month follow-up assessments so that the final sample would be 120 per arm. A total of at least 240 patients with HIV would have to be recruited for the study.

Descriptive statistics describing patient characteristics were represented in means, standard deviations (SD), medians and interquartile ranges (IQR), and percentages.

The primary outcome was compared between the intervention and control group by using a logistic Generalized estimating equations (GEE) model to account for correlated patient response [39]. A model investigating the proportion of abstinence/low alcohol use versus hazardous or harmful alcohol use was fitted.

Secondary outcomes (reduction in absolute AUDIT score, percentage decrease in AUDIT score, health related quality of life, depression score, stigma score, last measured CD4 cell count, last viral load, adherence to ART) were compared between intervention and control group using a linear mixed model [40], accounting for repeated measurements per patient and correcting for possible interviewer bias (added as random interviewer effect in the model). A logistic (GEE) model similar to the one used for the primary outcome was fitted to the secondary outcome tobacco use. Possible interviewer bias with respect to the administration of the intervention was also accounted for in the model by including a fixed baseline-interviewer effect. The full model contains all possible confounders (baseline: interviewer, intervention, age, audit score, gender, on ART, current TB-treatment, last CD4 cell count, therapy adherence, quality of life score for all domains, depression score and stigma score). Model building was done by removing covariates with the highest p-values (generally p>0.10) and performing a likelihood ratio test to ensure that the fit of the reduced model is not significantly different from that of the full model.

All statistical tests were two-sided and were considered statistically significant at α = 0.05. The GEE, MIXED, and GLIMMIX procedures of SAS version 9.4 (SAS Institute Inc., 2013) were used to fit the logistic GEE and mixed models.

### Compliance with ethical standards

Ethical approval was given by the Medunsa Research and Ethics Committee (Project number: MREC/H/165/ 4152011: IR). The Tshwane Research Committee has provided approval for the study (Project number: 2011/46). Procedures followed were in accordance with the ethical standards of the Medunsa Research & Ethics Committee and with the 1964 Helsinki declaration and its later amendments or comparable ethical standards (Duke C0784, UCT 032/2015). Public trial registration: Pan African Clinical trial Registry: PACTR201202000355384.

#### Informed consent

Written informed consent was obtained from all individual participants reincluded in the study.

## Results

### Patient characteristics

**Fig 1:**
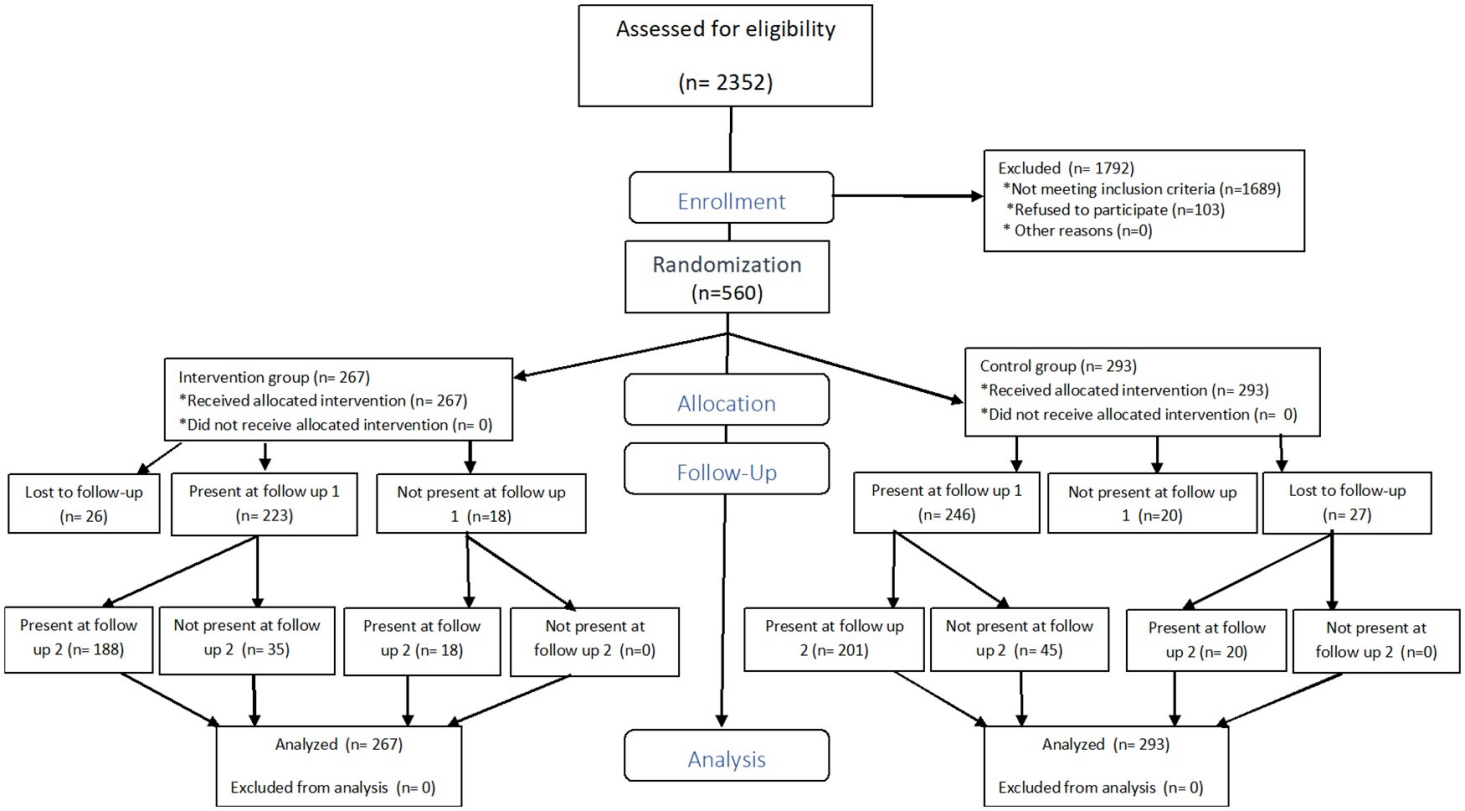
Flowchart of participants in the trial.

From January until June 2012, 2352 patients were approached to participate; 103 refused (response rate 95.6%) and 2249 were eligible and agreed to participate in the study (Fig 1).

The follow up period was up to December 2013. Of these 2249 eligible patients, 560 (24.9%) fulfilled the criteria for hazardous or harmful alcohol use and 267 patients (47.7%) were randomized into the intervention group and 293 (52.3%) in the control group (Fig 1). Table 1 gives the baseline characteristics of these patients. Most patients were male (53.9%) with a median age of 36 years (IQR 31–42), 84.8% were on ART for a median time of 21 months (IQR 6–39.5).

**Table 1:** Baseline characteristics.

Variable		Total (n = 560)	Intervention (n = 267)	Control (n = 293)	p-value
**Socio-demographic characteristics**					
Gender	Male	302 (53.9)	151 (56.5)	151 (51.5)	0.2
	Female	258 (46.1)	116 (43.5)	142 (48.5)	
Age^a^		36 (31–42)	36 (30–42)	36 (31–42)	0.8
Marital status	Never married	349 (62.4)	173 (64.8)	176 (60.3)	0.4
	Married/cohabiting	159 (28.4)	69 (25.8)	90 (30.8)	
	Separated/ divorced/ widowed	51 (9.1)	25 (9.4)	26 (8.9)	
Qualification	< Grade 7	71 (12.7)	29 (10.9)	42 (14.3)	0.2
	Grade 7–12	450 (80.4)	223 (83.5)	227 (77.5)	
	>Grade 12	39 (7.0)	15 (5.6)	24 (8.2)	
Income	Formal or informal salary	305 (54.5)	151 (56.5)	154 (52.6)	0.3
	Family contributions	183 (32.7)	79 (29.6)	104 (35.5)	
	Social grants/none/other	72 (12.9)	37 (13.9)	35 (11.9)	
Neighbourhood	Formal urban	449 (80.2)	215 (80.5)	234 (79.9)	1.0
	Informal settlement	71 (12.7)	33 (12.4)	38 (13.0)	
	Rural	40 (7.1)	19 (7.1)	21 (7.2)	
**Health characteristics**					
On ART		475 (84.8)	229 (85.8)	246 (84.0)	0.6
Duration on ART (months)^a^		21 (6–39.5)	19 (3–35)	23 (8.5–42)	0.03
Tobacco use		219 (39.5)	106 (39.8)	113 (39.1)	0.9
CD4 count at start ART^a^		168 (78–261)	163 (76–259)	171.5 (87.5–274.5)	0.5
Last measured CD4 count^a^		284 (183–413)	269.5 (175-5-352.5)	296 (185–459)	0.024
Ever tuberculosis		269 (50.6)	131 (51.2)	138 (50.0)	0.8
Currently TB treatment		32 (8.0)	20 (10.0)	12 (6.0)	0.1
WHO stage	1	64 (16.0)	25 (13.4)	39 (18.3)	0.6
	2	225 (56.4)	107 (57.5)	118 (55.4)	
	3	101 (25.3)	49 (26.3)	52 (24.4)	
	4	9 (2.3)	5 (2.7)	4 (1.9)	
NRTI	TDF	237 (42.8)	125 (47.2)	112 (38.7)	0.046
	AZT	103 (18.6)	46 (17.4)	57 (19.7)	0.5
	D4T	121 (21.8)	52 (19.6)	69 (23.9)	0.2
	LPV/r	13 (2.3)	6 (2.3)	7 (2.4)	0.9
Adherence VAS>95%	(if ART started)	315 (68.8)	145 (65.6)	170 (71.7)	0.1
Weight^a^		63.4 (56–72.8)	62.9 (55.9–72.6)	63.7 (56.2–73.0)	0.8
BMI^a^		22.2 (19.8–26.0)	22.0 (19.6–26.2)	22.4 (19.9–25.9)	0.7
**AUDIT-score**^b^			12.99 (3.00)	10.99 (2.87)	

Results in total number (percentage),

^a^Median (first-third quartile),

^b^Mean (Standard deviation)

The median duration of the interview was 40 minutes (IQR 30–47 minutes) in the intervention group (including the intervention) and 35 minutes (IQR 25–40 minutes) in the control group. Almost 91% of the patients in the control group were in the hazardous alcohol use group and 9.2% in the harmful alcohol use group, compared to 77.9% and 22.1% respectively of patients in the intervention group. Of patients receiving ART (84,8%), the duration being on ART was longer in the control group than in the intervention group. The last measured CD4 count was higher in the control group (Table 1). The median time between the baseline visit and visit 1 was 5 months and 12 months for visit 2. In the intervention arm 223 of 267 (83.5%) patients returned for visit 1 and 206 of 267 (77.1%) for visit 2. In the control arm 246 of 293 (83.9%) patients returned for visit 1 and 221 of 293 (75.4%) for visit 2.

### AUDIT score at baseline, time point 1 (5 months) and time point 2 (12 months) regardless of intervention

For the whole study group there was a significant decrease of around 6 points (SD = 0.27) in the absolute AUDIT score from baseline at both time points 1 (p<0.0001) and 2 (p<0.0001). The difference in AUDIT-score between time points 1 and 2 was not significant (0.451, SD = 0.30, p = 0.393).

### Effect of the intervention

**Fig 2:**
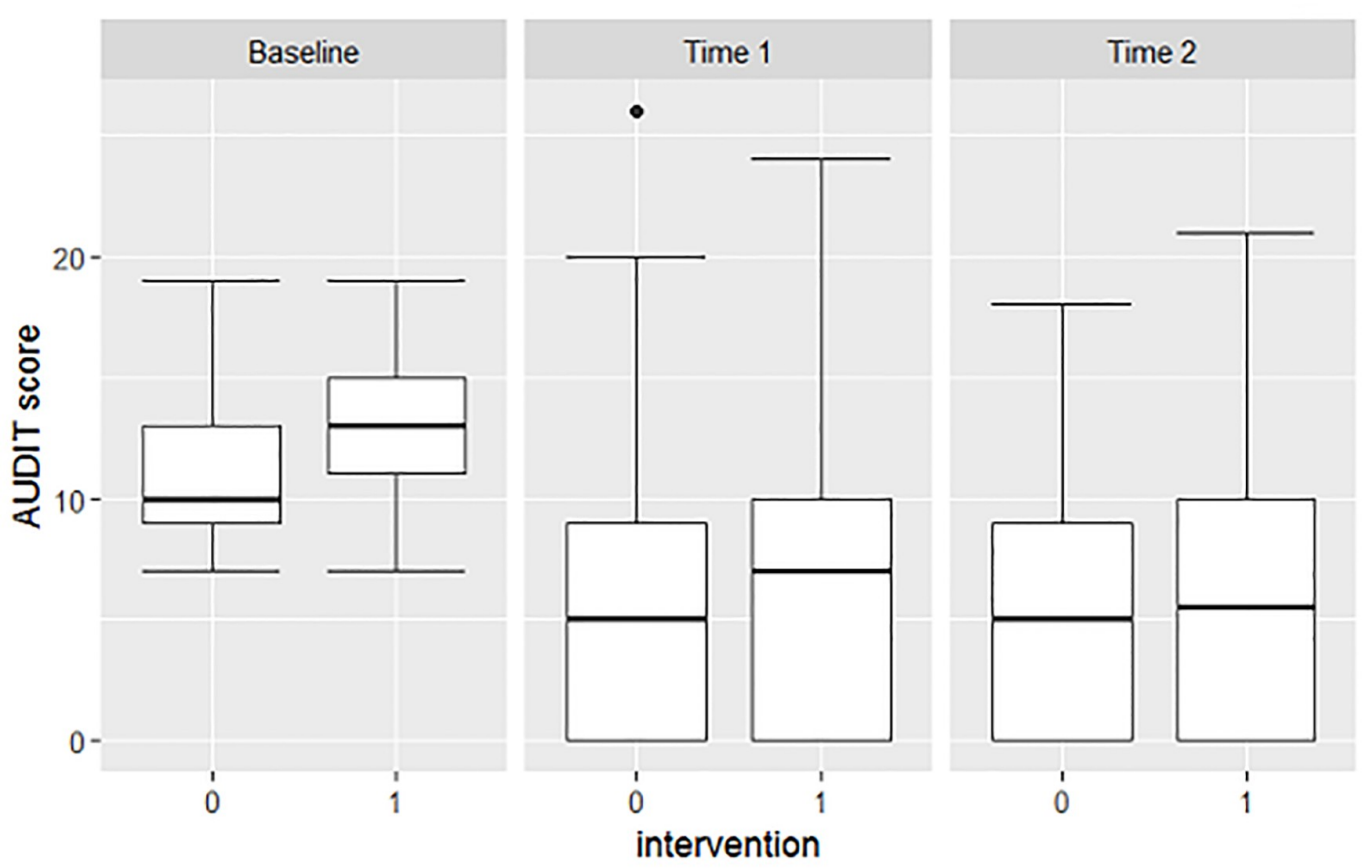
AUDIT score for the different intervention groups at different time points. AUDIT score for the different intervention groups measured at baseline and at time point 1 and at time point 2 after the intervention. Box represents first and third quartile of data. (0 = control, 1 = intervention).

In both the intervention and control group there was a significant decrease in AUDIT scores at follow up time points 1 and 2 compared to baseline (Fig 2, Table 2), but there was no significant decrease in AUDIT score between time points 1 and 2. The significant decrease in AUDIT score between baseline and the follow up time points was not different between the intervention and control group.

**Table 2:** Mean difference in AUDIT score (with 95% CI) within intervention and control group between different time points.

*Difference in AUDIT score*	*Intervention*	*p-value*^a^	*Control*	*p-value*^a^
Between baseline and time point 1	-6.388 (-7.533, -5.243)	<0.0001	-5.670 (-6.760, -4.579)	<0.0001
Between baseline and time point 2	-7.062 (-8.199, -5.925)	<0.0001	-5.899 (-6.994, -4.804)	<0.0001
Between time point 1 and time point 2	0.673 (-0.595, 1.942)	1	0.229 (-0.990, 1.449)	1

^a^ Bonferroni-adjusted p-value

### Reduction from hazardous or harmful alcohol use to alcohol abstinence or low risk alcohol use (primary end point; logistic GEE model)

Overall, around 58% and 61% of the patients had a reduction of alcohol use from hazardous or harmful at baseline to abstinence or low risk alcohol use at time points 1 and 2, respectively. Around half of the patients both in the control as the intervention groups reached the group of abstinence or low alcohol use at both time points (Table 3). Using the logistic GEE model, there was no significant difference between the intervention and control group in the odds ratio (OR) for alcohol abstinence or low risk alcohol use compared to hazardous or harmful alcohol use. On the other hand, regardless of the group, the odds of reduction of alcohol use from hazardous or harmful alcohol use to alcohol abstinence or low risk alcohol use at time point 1 was significant at 1.24 (95% CI 1.02–1.51) and for time point 2 it was 1.45 (95% CI 1.18–1.78). The nurses who performed the baseline interviews and carried out the intervention had a significant influence on the outcome. Indeed, the odds of alcohol abstinence or low risk alcohol use was 4.25 (95% CI 2.57–7.04) if interviewed with nurse 4 compared to nurse 1 and 1.68 (95% CI 1.11–2.54) if interviewed with nurse 4 compared to nurse 2.

**Table 3:** Number of patients at follow up in AUDIT groups.

		Baseline Total	Time point 1 Total (% of total patients at baseline)	Time point 2 Total (% of total patients at baseline)
**Total Number of Respondents**		**560**	**469**	**427**
Abstinence or low risk alcohol use group*	Control	0	153 (52.2)	142 (48.5)
	Treatment	0	117 (43.8)	118 (44.2)
	**Total**	**0**	**270**	**260**
Hazardous or harmful alcohol use group*	Control	293	93 (31.7)	79 (27.0)
	Treatment	267	106 (39.7)	88 (32.9)
	**Total**	**560**	**199**	**167**

*by AUDIT

### Difference in AUDIT score and percentage of change in AUDIT score (secondary end points; linear mixed model)

**Fig 3:**
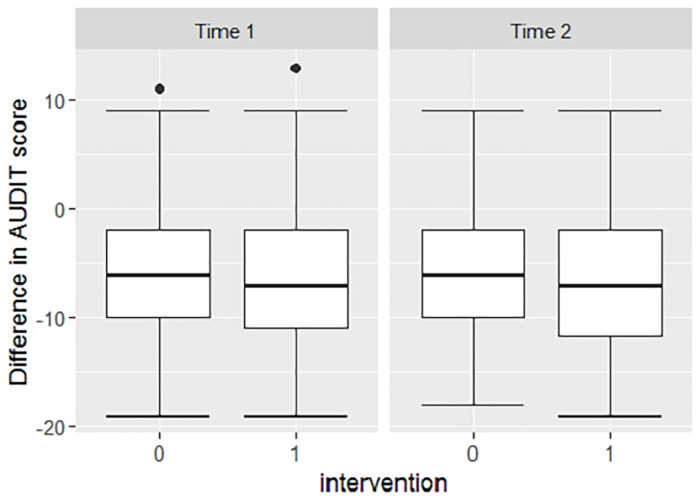
AUDIT score difference between baseline and time point 1 and baseline and time point 2 for the 2 groups. Box represents first and third quartile of data, 0 = control, 1 = intervention.

The difference in AUDIT score from baseline to first and to second follow up did not differ between the intervention and control groups (Fig 3). Being male, having started ART and having a higher QOL-domain 5 (environment domain) score reduced AUDIT score significantly more.

The percentage change in AUDIT score was not significantly different between the intervention and control group. Men (compared to women) and patients on ART (compared to patients not on ART) had a significantly higher percentage of change in AUDIT score. In patients with higher stigma scores at baseline, the percentage of change in AUDIT score was less than in patients with lower stigma scores.

### Effect of the intervention in medical related outcomes, quality of life outcomes, depression score and level of experienced stigma

The mean last measured CD4 count was significantly lower in the intervention group than in the control group at time point 1 (p = 0.036), but not at time point 2 (Table 4). The domain 3-QOL (level of independence) score was significantly higher in the intervention group (p = 0.014) (Table 4). The intervention had no influence on the quality of life outcomes, depression scores, stigma, tobacco use, viral load and therapy adherence at both time points. In all secondary outcomes, there was no significant interaction between intervention and time (Table 4).

**Table 4:** Intervention versus control in secondary outcomes.

Secondary outcome	Estimated intervention effect (95% CI)	p-value
Quality of Life	-0.001 (-0.102, 0.100)	0.986
Health Satisfaction	0.089 (-0.016, 0.193)	0.986
Domain 1	0.247 (-0.110, 0.604)	0.175
Domain 2	0.025 (-0.298, 0.349)	0.878
Domain 3	0.461 (0.095, 0.827)	0.014
Domain 4	0.302 (-0.063, 0.667)	0.105
Domain 5	0.190 (-0.032, 0.411)	0.093
Domain 6	0.134 (-0.033, 0.494)	0.086
Cesd10	-0.185 (-0.770, 0.400)	0.535
Stigma	-0.046 (-0.264, 0.172)	0.677
Tobacco Use	0.395 (-0.003, 0.793)	0.052
Last measured CD4 count	-0.153 (-0.296, -0.010)	0.036
Viral load	0.005 (-0.053, 0.062)	0.874
VAS	0.047 (-0.160, 0.253)	0.656

P-value estimates are based on a linear/logistic mixed model fitted to each secondary outcome. Intervention effect is adjusted for different baseline characteristics.

## Discussion

Since there is a high prevalence of high level alcohol use with risk for morbidity among patients with HIV there is a need for interventions to reduce alcohol use. In sub-Saharan Africa the health systems providing care to patients with HIV are overwhelmed by the large numbers of patients using the system. Possible interventions should be short and easy to implement in daily care provision. The brief intervention evaluated in this trial was based on an Information-Motivation-Behavioural Skills Model and was delivered once during a standard clinic visit. There was no difference in alcohol reduction in the intervention compared to the control group both 5 as 12 months after the counselling session. However, the alcohol use in both groups reduced significantly between baseline visit and visit 1 (after a median time of 5 months). There might be different explanations for this. Both the intervention as the control group received a health education leaflet on responsible drinking, emphasizing the idea of sensible limits. With both the baseline interview, covering questions about alcohol use and health, and the health education leaflet, the patients in the control group might also have been triggered to reduce their alcohol intake. This effect of alteration of study participants’ way of thinking about their alcohol use, only after assessment of it by interview or questionnaire, was recognized previously [41,42,43,44]. This “participation effect” might explain the reduction of alcohol use in the control group in our study. However, another randomized study conducted in rural Uganda studied assessment effects on alcohol use in patients with HIV, new to HIV care, who reported any alcohol consumption in the prior year [45]. One group was quarterly assessed (interviews, breath alcohol analysis and blood draws) versus another group that engaged in these procedures only at 6 months. At 6 months follow up there were no differences in unhealthy drinking in the 2 groups and thus no evidence was found for assessment reactivity. Another explanation for our outcome might be that with the stigma associated with alcohol use, a response bias might be likely due to social desirable answers. Underreporting of alcohol use has been confirmed in several studies comparing biomarkers for alcohol use to reported alcohol use [46,47].

There was no further reduction of alcohol use between time point 1 and time point 2; the reported alcohol use remained stable. These results are comparable to a study in Kampala in Uganda which also showed a significant decline in AUDIT-C score in both intervention as control arms in the 6 months follow up after a similar single brief alcohol reduction intervention session. In the latter study there also was no difference in both arms regarding alcohol reduction [23] but the control arm also received a set of standardized “positive prevention” information, including avoiding alcohol use. Most studies on alcohol reduction in Africa related to HIV focus on sexual risk reduction and are conducted in patients at high risk of contracting HIV [48]. There are few other studies in Africa focusing on alcohol reduction in people with HIV. Peltzer et al. tested a three-session (over a 4-month period) theory-based motivational-skills building risk reduction counselling intervention in patients in South Africa who just received a diagnosis of HIV [49]. The intervention contained, among other risk-reduction strategies aiming at increasing safer sex practices, information on alcohol use. The intervention was successful in reducing the overall use of alcohol and alcohol use related to sex at 4 months [49]. A study conducted in Kenya among HIV-outpatients consisting of a culturally adapted six-session (weekly, 90 minutes sessions) gender-stratified group cognitive-behavioral therapy intervention, showed at 30-day follow up a significant reduction in percentage of drinking days and mean drinks per drinking day in the intervention group. Reported alcohol abstinence at the 90-day follow-up was 69% compared to 38% (standard of care-group) [50]. Zule et al. reported an higher abstinence-rate from alcohol at 12 months in women with HIV in South Africa after an intervention consisting of two sessions of 1 hour focusing on alcohol and drugs use, sexual risk behaviour and empowerment of the women compared to an intervention on nutritional information and a standard-of-care group [51]. It is difficult to compare the results of these three intervention studies to our study. First the study-groups differ, including only patients who just receive a diagnosis of HIV, or only women with HIV compared to an unselected group of patients with HIV in an outpatient HIV clinic in our study. The focus of intervention (focusing on more issues than only alcohol reduction in the other studies), the setup of the sessions (frequency, duration,…), the follow-up time and the person delivering the intervention was different among the studies. However, all studies were successful in reducing alcohol use among the participants (at least for a short follow up time). In overcrowded clinics with limited consultation time per patient prevention of harmful alcohol use may not be a priority. However, it might be that a few short questions or messages or a leaflet on alcohol use could be effective in reducing alcohol use in persons with HIV. And it might be necessary to repeat this strategy over a follow up time to reinforce the alcohol reduction. This is a subject for further study.

In our study there was a significant higher reduction in percentage and absolute AUDIT score in men compared to women. This is in contrast with the study in Uganda that showed the opposite effect [23]. In our study the groups were not stratified for gender, so more men than women were included in the intervention group (151 versus 116 respectively), with a higher mean AUDIT score in men (13.5, 95% CI 13.0–14.0) than in women (12.3, 95% CI 11.8–12.8) and with more men being in the harmful alcohol use category (27.8%) versus women (14.7%); and 72.2% of men being in the hazardous alcohol use category versus 85.3% in women. In other studies men were shown to do better during brief alcohol interventions [38], while women show more assessment reactivity and therefore female control groups show improvements which mask the effects of intervention [52]. It should also be taken into account that criteria for normal alcohol use are more strict in women (score of ≤6) than in men (score ≤7). This however does not fully explain the difference found between men and women in reduction in AUDIT score in our study and requires additional investigations.

The reduction in alcohol use in both groups still remains significant and important for health outcomes. The risk of all-cause mortality, cancers and high blood pressure rises in the general population with increasing levels of alcohol consumption[53,54]. Moreover, the level of consumption that minimises health loss is zero [53]. So the aim should be abstinence of alcohol use, but any reduction could have an impact on health (both physically as mentally), but also on social-, family- and work life.

There are several possible limitations in this study. There was a difference in AUDIT-score between the intervention and control group at baseline, with a higher mean score in the intervention compared to the control arm. However, because randomisation was done, any observed differences at baseline is attributed purely by chance and no conclusion from this can be derived [55]. A limitation of the study was that there was no blinding, neither for study nurses nor for statisticians, and the lack of a biomarker to assess the effect of the intervention. Blinding was intended, as described in the original study protocol, with nurses switching from one clinic to the other after inclusion. However, the inclusion period was longer than intended, giving an overlap of inclusion- and follow up points (whereby the nurses recognized the patients and the group they were randomized into). Also, patients did not show up for follow up themselves, but the nurses had to recognise them in the waiting room and ask if they wanted to come for a follow up interview. Moreover, the nurse applying the interview and brief intervention significantly affected the outcome (significant interviewer effect inducing social desirable results), and the nurses may also have increased the fidelity to the intervention which implies an intervention bias. However during monitoring visits no violations of the study protocol were picked up.

Patients in the intervention group had a lower last measured CD4 cell count at baseline and at time point 1. Data on influence of (heavy) alcohol use on CD4 cell count is contrasting, in our previous report of the whole study group (n = 2230) there was a significant lower last measured CD4-cell count in high risk drinkers versus low risk drinkers (350 versus 309 cells/μL, p = 0.0012) [1], but less high risk drinkers than low risk drinkers started ART (83.9% versus 90.3%, p = 0.000) and when on ART, they were less adherent to therapy (ART VAS ≥95%: 67.3% versus 82.7%). A study in Kampala showed that alcohol use was shown not to have a direct effect on CD4 count levels but CD4 count levels may decrease in persons with alcohol use because of non-adherence with the ART [56].

Finally, the study was conducted in three primary HIV clinics in one region in South Africa and therefore results might not be generalizable to other settings.

## Conclusion

A brief alcohol intervention, based on an Information-Motivation-Behavioural Skills Model and delivered once during a standard clinic visit, was not more successful at reducing alcohol use both at 5 and 12 months after the intervention, compared to only the administration of a questionnaire on alcohol use and the distribution of a health educational leaflet on responsible drinking. However, there was a beneficial effect on reported hazardous or harmful alcohol use at least over a short term follow up period with both interventions. It might be that only an interview and/or a health leaflet can be successful in reducing alcohol use but this needs to be investigated with more objective measures of alcohol use. To sustain an effect most likely repetitive contacts with harmful alcohol drinkers will be needed during a long follow up period.

## Supporting information

S1 File CONSORT 2010. Checklist of information to include when reporting a randomised trial.
(PDF)

S2 File Trial protocol. Screening and brief intervention for alcohol problems in HIV outpatients in the clinics associated with Dr. George Mukhari Hospital, Ga-Rankuwa, South Africa: a single-blinded randomized controlled trial.
(DOCX)
