# Supplementary material for: The efficacy of a brief intervention to reduce alcohol use in persons with HIV in South Africa, a randomized clinical trial
Source: PLoS One. 2019 Aug 20;14(8):e0220799. doi: 10.1371/journal.pone.0220799 (PMC6701742; doi:10.1371/journal.pone.0220799)
Supplement: S2 File — Screening and brief intervention for alcohol problems in HIV outpatients in the clinics associated with Dr. George Mukhari Hospital, Ga-Rankuwa, South Africa: a single-blinded randomized controlled trial. (DOCX) [file pone.0220799.s002.docx]

Screening and brief intervention for alcohol problems in HIV outpatients in the clinics associated with Dr George Mukhari Hospital, Ga-Rankuwa, South Africa: A single-blinded randomized controlled trial.

Substudy for the study:

Screening and Brief Intervention for Alcohol problems in Dr George Mukhari Hospital out-patients, South Africa: a single-blinded randomized controlled trial.

Research proposal

PI: Diana Huis in ’t Veld, MD, PhD Student University of Antwerp, Belgium

Co-PI: Prof Supa Pengpid, Dr. PH: University of Limpopo, South Africa

Co-PI: Prof Karl Peltzer, PhD: Human Science Research Council, South Africa

Promotor Diana Huis in ’t Veld: Prof R. Colebunders, University of Antwerp

Part of VLIR Project 7: Public Health Intervention

Table of Contents

[Background 4](#_Toc296460396)

[Problem statement 6](#_Toc296460397)

[Purpose/Aim/Objectives 6](#_Toc296460398)

[Aim 6](#_Toc296460399)

[Objectives: 6](#_Toc296460400)

[Hypotheses: 6](#_Toc296460401)

[Methodology 7](#_Toc296460402)

[Design 7](#_Toc296460403)

[Study population and participants 7](#_Toc296460404)

[Inclusion criteria 7](#_Toc296460405)

[Exclusion criteria 7](#_Toc296460406)

[Sample size calculation 7](#_Toc296460407)

[Procedure 7](#_Toc296460408)

[Data collection instruments 8](#_Toc296460409)

[Questionnaire and screening test (Annex 1) 8](#_Toc296460410)

[Demographic Characteristics 8](#_Toc296460411)

[Sexual behavior 9](#_Toc296460412)

[Health and quality of life related questionnaires (annex 2) 9](#_Toc296460413)

[Health-related quality of life (QoL) 9](#_Toc296460414)

[Depression 9](#_Toc296460415)

[Internalized AIDS stigma 9](#_Toc296460416)

[Alcohol consumption (annex 3) 9](#_Toc296460417)

[HIV related information Data Extrect Sheet (Annex 4) 10](#_Toc296460418)

[Assessment of adherence to ART (Annex 5) 10](#_Toc296460419)

[Visual Analog Scale (VAS) 10](#_Toc296460420)

[Data from pharmacy: 10](#_Toc296460421)

[Pill count 10](#_Toc296460422)

[Prescription refill 11](#_Toc296460423)

[Reliability, validity and objectivity of the data collection instruments 11](#_Toc296460424)

[Interventions 11](#_Toc296460425)

[Experimental arm: 11](#_Toc296460426)

[Brief intervention 11](#_Toc296460427)

[Control arm: 11](#_Toc296460428)

[Counselor Training and Intervention Quality Assurance 12](#_Toc296460429)

[Data Analysis 12](#_Toc296460430)

[Ethical issues 12](#_Toc296460431)

[Adequacy of Protection against Risks 13](#_Toc296460432)

[Confidentiality 14](#_Toc296460433)

[Dissemination of research findings: 14](#_Toc296460434)

[Time line and research activity 15](#_Toc296460435)

[Budget 16](#_Toc296460436)

[Annexes 17](#_Toc296460437)

[Annex 1: Patient interview schedule and Demographic Characteristics 17](#_Toc296460438)

[Annex 2: Health-related quality of life (QoL) 19](#_Toc296460439)

[Quality of life 19](#_Toc296460440)

[Depression: 20](#_Toc296460441)

[Sexual Behaviour 21](#_Toc296460442)

[Internalized AIDS stigma 21](#_Toc296460443)

[Annex 3: Alcohol consumption 22](#_Toc296460444)

[Patient interview schedule and Demographic Characteristics 24](#_Toc296460445)

[Health-related quality of life (QoL)-Setswana 25](#_Toc296460446)

[Quality of life-SeTswana 25](#_Toc296460447)

[Depression-Setswana 26](#_Toc296460448)

[Sexual Behaviour-Setswana 27](#_Toc296460449)

[Internalized AIDS stigma-Setswana 27](#_Toc296460450)

[Alcohol consumption-SeTswana 28](#_Toc296460451)

[Annex 4: Patients Information Extract Sheet: HIV related information 31](#_Toc296460452)

[Annex 5. Assessment of adherence to ART (compiled by the Pharmacy assistant) 33](#_Toc296460453)

[Visual Analog Scale (VAS) 33](#_Toc296460454)

[Pill count and Prescription refill 33](#_Toc296460455)

[Annex 6: Health education leaflet 37](#_Toc296460456)

[Annex 7: Consent form 39](#_Toc296460457)

[INFORMATION SHEET: STEP 1 39](#_Toc296460458)

[CONSENT FORM: STEP 1 41](#_Toc296460459)

[INFORMATION SHEET: STEP 2 42](#_Toc296460461)

[CONSENT FORM: STEP 2 43](#_Toc296460462)

[Annex 8: Consent form Tswana 45](#_Toc296460463)

[INFORMATION SHEET: STEP 1 45](#_Toc296460464)

[FOROMO YA TETLELELO: LENANEO LA NTLHA 47](#_Toc296460465)

[LETLAKALA LA KITSOKAKARETSO: LENANEO LA BOBEDI SOUTH AFRICA HEALTH RISK INTERVENTION 48](#_Toc296460467)

[FOROMO YA TETLELELO: LENANEO LA BOBEDI 49](#_Toc296460468)

[References 50](#_Toc296460470)

# Background

The use of alcohol in South Africa is among one of the highest of Africa^1^, with an adult per capita consumption of 9.5 liters of pure alcohol per year (2003-2005 period)^2^. However, if only taking into consideration the people that drink alcohol, the total adult per capita consumption is 34.91 liters of pure alcohol per year (men 39.64, women 23.84 liters)^2^. Since South Africa also has one of the highest prevalence of HIV in the world with a current prevalence estimated to be 17.8% [17.2%-18.3%]^3^ it is possible that a considerable number of HIV positive people consume alcohol.

Studies from Southern and Eastern Africa showed that in adults who have a history of alcohol consumption or frequently use alcohol, the HIV prevalence is considerably higher than in adults that do not use alcohol.^4,5,6,7^ Percentages range from 10% in individuals who ever drank alcohol compared to 5% of individuals who never drank alcohol^8^, to 58.6% in women drinking alcohol in a township in South Africa compared to 34.7% of women who never drink alcohol^9^. Individuals that drink alcohol have an increased risk of HIV infection compared to adults with no history of alcohol consumption (odds up to 2.5 times higher)^10^. A systematic review and meta-analysis by Fisher et al. confirmed the strong association between alcoholuse and HIV infection^11^.

It has been shown that alcohol use in HIV positive patients has numerous negative effects. An association has been shown between alcohol use and high-risk sexual behavior and risk of HIV^12,13^. Participation in pretest counseling among women attending PMTCT services was significantly higher in women with a low alcohol score compared to women with a high alcohol score (Odds ratio (OR)=0.71). Also posttest counseling participation was higher in women with low alcohol score (OR=0.65)^14^. Alcohol use was a predictor of failure to return for HIV test results among pregnant women in Tanzania. In women using alcohol daily the adjusted OR (aOR) was 1.24 (95% CI: 0.41-3.77) and 1.55 (95% CI: 1.06-2.25) for women drinking alcohol occasionally^15^. Alcohol use was associated withlate presentation to HIV care (OR 3.55, 95% CI: 1.63-7.71)^16^.

Alcohol use in HIV patients also has negative effects on adherence. In a study from Botswana alcohol use influenced 37.0% (n=148) patients to miss ART doses^17^. It was also associated with poor adherence in a study from India (adjusted OR 5.68)^18^, West-Africa(OR 1.4, CI 1.1-2.0)^19^, the Democratic Republic of Congo (17 out of 44 patients (39%) who reported to have missed ART>2 consecutive days during the last month report to drink at least more than 1 alcoholic drink a day compared with 491 of 1909 (26%) who did not report missed antiretroviral doses > 2 consecutive days (OR=1.82, 95% CI: 0.94 to 3.49)^20^ and in a study looking at adherence in 5 African countries^21^.

HIV disease progression is accelerated by alcohol use as shown in different studiesin which frequent alcohol users (defined by 2 or more drinks daily) were 2.91 times (95% CI: 1.23-6.85) more likely to present a decline of CD4 to ≤200 cells/µL, independent of baseline CD4 cell count and HIV viral load, ART use over time, time since HIV diagnosis and age and gender. Frequent alcohol users who were not on ART also had an increased risk for CD4 cell decline to ≤200 cells/µL (HR=7.76, 95% CI 1.2-49.2)^22^. A study by Samet et al. showed that heavy alcohol consumption was associated with a lower CD4 cell count (adjusted mean decrease of 48.6 cells/µL compared with abstinence; p=0.03) in patients who were not on ART. In patients on ART there was no association found between heavy alcohol consumption and lower CD4 cell count.^23^

Conigliaro and colleagues showed that patients with alcohol diagnoses more often had elevated alanine transaminase or aspartate transaminase levels (p≤0.02) (which could add to the risk of toxicity), anemia (p<0.001) and elevated mean corpuscular volume (P<0.001). Also hazardous and/or binge drinkers more often had a detectable viral load (P<0.001)^24^. Finally, a method to quantify the fraction of HIV/AIDS deaths attributable to alcohol consumption from non-adherence to ART shows that the effects of alcohol on HIV/AIDS in the African Global Burden of Disease regions range from 0.03% to 0.34% for men and from 0% to 0.17% for women, depending on region and age category. The detrimental effect of alcohol consumption was statistically significant in every region and age category except for the North Africa/Middle East region.^25^

Very few reports from resource limited settings are published on interventions to reduce alcohol use in HIV positive patients. One study describes an intervention based on culturally adapting 6 session (over a 3 month period) cognitive-behavioral therapy (CBT) to reduce alcohol use among HIV-infected outpatients in Western Kenya, whereby percent days abstinent from alcohol (PDA) before session 1 was 52-100% (women) and 21-36% (men). By session 6 this was 96-100% for women and 89-100% for men. PDA effect sizes (Cohen’s d) between first and last CBT session were 2.32 (women) and 2.64 (men). Participants were also satisfied with the treatment^26^. Another intervention set in Zimbabwe aimed to reduce the sexual risk behavior associated with men’s alcohol consumption and was evaluated in a randomized controlled trial implemented in 24 beerhalls. It was a male-focused, peer-based intervention promoting the idea that men can assist their friends in avoiding high-risk sexual encounters associated with alcohol drinking. The levels of risk behavior declined in both the control as intervention arm. The intervention did not add an impact on the alcohol dependency (the primary outcome measure; episodes of unprotected sex with non-wife partners in the preceding 6 months; also did not differ in the two groups)

# Problem statement

Alcohol use and HIV are closely related and the consequences for individuals with HIV infection who use alcohol are negative in a broad spectrum, including increased sexual risk behavior and psychosocial problems (depression). Since the combined occurrence of epidemics of HIV and alcohol use has been found to be a major problem in South Africa there is a need for action to reduce alcohol use among HIV positive patients. A randomized controlled trial is proposed aimed at evaluating Screening and Brief Intervention (SBI) for Alcohol problems among HIV out-patients in the clinics under the Dr. George Mukhari Hospital, Ga-Rankuwa, South Africa.

# Purpose/Aim/Objectives

#### Aim

To study the efficacy of a Screening and Brief Intervention (SBI) for alcohol problems-project in HIV-positive out patients.

#### Objectives:

- - 1. To measure the prevalence of alcohol consumption among HIV positive hospital outpatients;
    2. To describe drinking patterns, health status, quality of life and sexual behavior among HIV positive out-patients, and identify medium risk drinkers needing intervention;
    3. To compare level of alcohol consumption, health status, quality of life, level of adherence to ART and sexual behavior among medium risk drinkers within the previously identified group between pre intervention, 3 months and 12 months after intervention;
    4. To compare **levels of** (a) alcohol consumption, (b) adherence level to ART, (c) quality of life, (d) health status and (e) number of patients with virological and immunological failure, between intervention and control group at 3 and 12 months;
    5. To compare **changes in** (a) alcohol consumption, (b) adherence level to ART, (c) quality of life, (d) health status and (e) number of patients with virological and immunological failure, between intervention and control group at 3 and 12 months.

#### Hypotheses:

- HIV positive patients who are medium risk drinkers in the intervention group decrease drinking more than those in the control group;
- The levels of alcohol consumption of patients in the intervention group will continuously reduce over a 12 month assessment period;
- In the intervention group, adherence levels to ART during follow up will be higher, alcohol consumption lower, quality of life and health status better and number of therapies failing virologically or immunologically will be lower compared to the control group at post intervention;

# Methodology

#### Design

The study design for this efficacy study is a randomized controlled trial with 3 and 12-month follow ups.

#### Study population and participants

The sample will include HIV positive out-patients at the primary care clinics in the area. HIV positive out-patients will be screened for alcohol problems, and those identified as medium risk drinkers will be randomized into an intervention or control group.

#### Inclusion criteria

-HIV 1 positive male and female patients

-Outpatients

-18 years and above

-No mental impairment

-Medium risk drinkers (i.e. 8-19 for men and 7-19 for women on the AUDIT questionnaire)

#### Exclusion criteria

-A score of 20 and above on the AUDIT questionnaire

-A score of less than 8 for men and less than 7 for women on the AUDIT questionnaire

-Pregnant patients

-Patients already under alcohol reduction treatment

-Patients with mental impairment

#### Sample size calculation

The sample size was calculated using Open Epi (available on www.openepi.com), for a two arm randomized controlled trial. With 80% power, a significance level of 5%, two sided-tests, assuming that adherence levels to ART overall are 90% in Africa. The adherence level of moderate alcohol users compared to abstainers and minimal alcoholusers shows an odds ratio of 0.480^27^. The minimum sample size for each group is 251 patients. To accommodate a drop-out rate of 10% overall, the sample size for each group will be 276, making a total of 552 patients.

#### Procedure

The procedure follows the procedure of the main protocol.

Universal screening of all presenting HIV out-patients will be done. All consecutive HIV positive clients visiting the out-patient department will be screened for alcohol problems and randomized into an intervention or control group. Randomization will be done by a remote computer to generate numbers for intervention and control. Research assistant 1 will ask for consent from patients attending the hospital out-patient department to participate in the study, i.e. do a baseline assessment using the AUDIT questionnaire. Research assistant 1 will not be involved in delivering treatment. Research assistant 2 will score the results of the alcohol test section of the questionnaire. Clinic out-patients who score 8-19 for men and 7-19 for women on the AUDIT questionnaire after screening (risky drinkers) will be included in this study. Patients with a score of 20 and above on the AUDIT (with probable alcohol dependence) will be referred for further management. Additional information of the patients will be collected using the data collection instruments. Research assistant 2 will then check the random numbers which allocate the case to the intervention arm or control arms. Research assistant 2 will carry out the intervention for all the participants, after which they will be followed up at 3 and 12 months (which are standard visit intervals for the follow up of the HIV care), and assessments will be done by Research assistant 1, who will be blinded to the intervention allocation of the participants. In the event of a drop-out, at least six individual attempts will be made to contact patients by telephone and letter. Even if a contact was not successful at either follow up point, further attempts will be made at any next follow up point. Sampling will occur throughout all hours of clinic operation over a 3-month period. Two-hundred and seventy six patients will be recruited from hospital out-patient departments. Patients will be offered a drink (soda, water) during the interviews.

# Data collection instruments

Three instruments will be use to collected data: i) questionnaire and screening test, ii) patients information extract sheet, and iii) Assesment of adherence to ART by VAS and pill count form in the pharmacy.

#### Questionnaire and screening test (Annex 1)

###### Demographic Characteristics

A researcher-designed questionnaire will be used to record information on participants’ age, gender, educational level, marital status, income, and residential status. This questionnaire will be used at baseline. At every follow up visit changes will be checked briefly.

######

###### Sexual behavior

To study the sexual behavior of HIV patients who consume moderate amounts of alcohol before and after intervention, we included a questionnaire with questions on sexual behavior. This questionnaire will be administered at baseline and every follow up visit.

#### Health and quality of life related questionnaires (annex 2)

###### Health-related quality of life (QoL)

The WHOQoL-HIVBREF is based on the WHOQOLHIV measure, one of the two World Health Organization’s (WHO) QoL instruments for the use with HIV infected populations^28,29^. This questionnaire will be taken at baseline, and every follow up visit.

###### Depression

We will assess depressive symptoms using the 10-item version of the Centers for Epidemiologic Studies Depression Scale (CES-D)^30^. The CES-D has been widely used in studies of the relationship between HIV and depression^31^. The sensitivity and specificity of the CES-D 20-item survey has been reported to average 80% and 70%, respectively, compared to formal diagnostic interview^32^. We will also identify patients who experience more severe depressive symptoms by distinguishing those scoring greater or equal to 15 out of 30 on the CES-D 10-item survey^30^. This questionnaire will be taken at baseline and every follow up visit.

###### Internalized AIDS stigma

We will use the seven-item internalized AIDS-related stigma scale for people infected with HIV. Items reflect self-defacing beliefs and negative perceptions of people living with HIV/AIDS^32^. In previous studies with the same scale, a Cronback a reliability coefficient of 0.72-0.76 was found^33^. This questionnaire will be taken at baseline and every follow up visit.

#### Alcohol consumption (annex 3)

The 10-item Alcohol Disorder Identification Test (AUDIT)^34^ assesses alcohol consumption level (3 items), symptoms of alcohol dependence (3 items), and problems associated with alcohol use (4 items). Responses to items on the AUDIT are rated on a 4-point Likert scale from 0 to 4, with a maximum score of 40 points. AUDIT scores higher than 19 indicate more severe levels of risk; scores of 8-19 in men and 7-19 in women indicate a tendency to problematic drinking.

To reduce the stigma of alcohol use, WHO suggests to integrate the screening of alcohol use with screening of other health related behaviors. For this reason two questions will be asked about the use of tobacco products and anthropometric measurements will be taken to assess the riskfactor of overweight (height, weight, waist- and hip circumference)

This questionnaire will be administered at baseline, 3 and 12 months follow-up visits. All questionnaires will be administered in English or Tswana, the two languages predominantly spoken by nearly all clinic patients.

#### HIV related information Data Extract Sheet (Annex 4)

A researcher designed patient information extract sheet will be used to record information related to the HIV infection and will include questions about date of first positive HIV test, start date ART, ART regimen and co-infections. The patientfile will be used to obtain additional information. The computerized laboratory data system will be used to obtain the laboratory results. At follow up CD4 cell counts and viral load will be obtained from the computerized laboratory data system. This data extract sheet will be taken at baseline and laboratory and clinical information will be collected at every study visit; at baseline, 3 and 12 months.

#### Assessment of adherence to ART (Annex 5)

###### Visual Analog Scale (VAS)

The 30-day visual analog scale (VAS) provides an overall adherence assessment for a one-month period. The VAS has been validated in resource-limited settings^35,36^. Adherence levels assessed from the VAS are defined as follows: full adherence=100%, partial adherence: between ≥95% and <100%; and non-adherence as <95% of prescribed doses taken in the past 30 days. The VAS will be assessed at baseline for patients who are already on ART and on any follow up visit if the patient is on ART.

###### Data from pharmacy:

###### Pill count

Unannounced home based pill counts are a reliable means to estimate adherence, but are limited by costs and logistical feasibility. Kalichman et al. ^37^ ^[[1]](#endnote-1)^ showed that a high degree of concordance was observed between phone- and home-based number of pills counted (Intraclass Correlation, ICC=0.997, 95% CI 0.995-0.998, P<0.001). The concordance between pill counts was not influences by participants’ educations or health literacy. The adherence determined by telephone-based pill counts also corresponded with patient viral load, providing evidence for criterion-related validity.

Practical: The patient is called by phone by research assistant and asked to put all the pills on flat surface. The patient is asked to read the medication label and to count the pills. This is repeated for all the medication. The telephonic pill counts will be repeated at baseline at all follow up assessments. Since the pill count is unannounced, it can take place up to 2 months after the patient has been seen by the research nurse for assessment.

To compare the number of pills that were dispensed and that are remaining at the pill count. The following formula gives the % adherence:

| Did the client return the medication containers?  NO  YES  $\% Adherence= \frac{Dispensed-Returned}{Expected to be taken} X 100$ |
| --- |

# Reliability, validity and objectivity of the data collection instruments

All instruments to be used in this project are standard instruments, with proven content validity and reliability, with Cronback alpha greater than .80 for each set.

# Interventions

#### Experimental arm:

###### Brief intervention

Participants who are randomized in the intervention arm receive personalized feedback on their AUDIT results, a health education leaflet (Annex 6), plus brief counselling about reducing excessive drinking, during a one session 20 minute intervention.

The steps of brief counselling are: 1) Identify any alcohol related problems mentioned in the interview, 2) Introduce the sensible drinking leaflet, and emphasise the idea of sensible limits, and make sure that patients realize that they are in the medium-risk drinking category, 3) Work through the first 3 sections of the problem solving manual while mentioning the value of reviewing the other sections, 4) Describe drinking diary cards 5) Identify a helper, and 6) mention the 3, 6 and 12 months follow-up. The brief intervention will be based on the WHO brief intervention package for hazardous and harmful drinking^36^. The Information-Motivation-Behavioural Skills (IMB) Model

will be used to guide the alcohol reduction intervention in South Africa.

###### Control arm:

Participants randomized to this group will not receive feedback on the initial alcohol screening. They will be provided with a health education leaflet on responsible drinking (Annex 6), and they will only receive the brief intervention after the 12 month follow-up if the intervention has proved to be efficacious.

###### Counselor Training and Intervention Quality Assurance

The intervention research assistant will deliver the interventions to patients as per usual clinic services. There is a comprehensive manual for the brief intervention which will be used to guide the research assistant counselor throughout the session. The research assistant counselors had been trained to administer the intervention protocol through role playing and general skills training techniques in a 5 day workshop for the earlier trial, and will receive two additional days training for HIV and sexual related questions. Site visits will be done bi-weekly by one of the investigators and project members to offer support and supervision to the trained research assistant counselors.

# Data Analysis

*Definition of virological failure:*

We follow the definition used in the national guidelines in which virological failure is defined as a repeat VL ≥ 1000 copies/ml after intense adherence assessment^38^.

*Definition of immunological failure:*

Immunological failure is defined according to the WHO definition. Failure is defined as a fall of CD4 count to pretherapy baseline (or below), *or* a 50% fall from the on-treatment peak value (if known) *or* a persistent CD4 levels below 100 cells/mm^3^.^39^

Means, standard deviations, and percentages will be used for descriptive statistics. T-test for continuous data and chi-square for categorical data will be used to examine differences between groups. Generalized Linear Model Repeated Measures 2 x 3 Analysis of Variance (ANOVA) will be used for comparing observations (alcohol use score) across the three contact periods to demonstrate a treatment intervention x time interaction.Observations with a single follow-up point missing (at either 3 and 12 months) will be imputed with the available follow-up. Data for participants who are lost to follow-up at 3 and 12 months will be imputed using baseline values. SPSS for Windows version 18.0 (SPSS, Inc., Chicago, IL) will be used for calculations.

# Ethical issues

This study will not be conducted until ethical clearance is obtained from the Medunsa Research Ethics Committee. Since the sources of research data are participants’ responses to study measures, there are potential risks to human subjects. These include negative consequences if confidentiality of information obtained in the study (including subject identity as a research participant) were compromised; embarrassment, discomfort, or distress in response to the interviews discussing substance use, and frustration in completing assessment measures.

While the risks of administering the behavioral and demographic data questionnaire to the study participants of this study are considered minimal, steps will be taken to reduce this risk further by employing specially trained and supervised research assistants who will know how to prevent or handle any possible negative consequence.

#### Adequacy of Protection against Risks

Informed Consent: Research staff will conduct the informed consent process in a private room located in the area of the clinic. A research staff person will sit with the participant and work through the informed consent form together. After a detailed description of all study goals, requirements and risks are presented to eligible participants; written consent will be obtained prior to their enrolment into the study. Informed consent forms will be given and explained in the preferred language of the participant (English-Annex 7, or seTswana-Annex 8). The consent process is expected to take 10 minutes. Participants can take as long as they wish to decide whether or not to participate.

Voluntariness: All participation in this study will be voluntary. Participants may stop the data collection process at any time for any reason. Refusal to take part in this project will have no bearing on services at the health facility; all individuals, whether they agree to participate or refuse, will continue to receive treatment for their illness based on established standard of care. This will be made clear to the interviewees both before and during the consenting process.

Accountability of researchers: The names of the investigators will be included on all consent forms with phone numbers and addresses for the participants to use if they have any questions.

Data monitoring and protection: The researcher will be responsible for data management. All data forms and records collected during this research will be held in a secure location at the University of Limpopo for the duration of the proposed research. Confidentiality of all respondents will be ensured through the replacement of any personal information with unrelated unique identifiers. Wherever relevant, names and location information will be separated from the electronic data that are processed for analysis. The only identifiers used during the analysis will be a unique identification number.

Potential benefits: Essentially the participants may benefit from an increased awareness of their own alcohol risk behaviors and be empowered to do something about it. They will also learn in the experimental group from the intervention about how to reduce risky drinking.

While the immediate benefits of the survey questionnaire to individuals will be minimal, the information disseminated during this study will assist greatly with alcohol intervention program planning and implementation. The community as a whole will benefit from these data which will assist public health and clinical workers in the targeting of efforts to control alcohol problems in the area. The risk to the study subjects is minimal and reasonable, given the health risks associated with alcohol.

All participants in this study will be persons with medium risk drinking. It is anticipated that participants in the intervention will acquire knowledge and skills, and will receive support needed to reduce their alcohol risks. All participants will receive the intervention. In addition to the direct benefits associated with participation, this research is expected to yield important new information on intervention models for reducing alcohol risk behaviours in South African hospital patients. All field staff will be extensively trained in risk management and human participant protections.

#### Confidentiality

All research data obtained from participants will be labeled with a code number and not the participant’s name. Only the code number will appear on measures, data records, and computer files.

Obtaining Consent

Consent to participate will be obtained in a 2-stage process. Research assistants will initially ask for informed consent to conduct health screening and collect some basic demographic information and check eligibility to take part. No identifiable information will be collected at this stage. Patients who then are positive on the AUDIT (alcohol medium risk score), as applicable, will have the study explained to them verbally by another research assistant counselor and in writing (using the patient information sheet, annex 7-8). Informed consent will be obtained at this second stage which will include permission to give the patient's data and contact details to the research staff, and participate in the intervention and follow up after 3 and 12 months (see Annex 7-8: Consent form).

# Dissemination of research findings:

Results from the outcome evaluation will be disseminated through peer reviewed journal publications, published reports and presentations for dissemination to the university and district departments of health as well as presentations at relevant local meetings and conferences. In terms of feedback for hospital staff a summary sheet of the findings will be prepared and a full report will be made available.

# Time line and research activity

| **Research Activity** | **Month/Year** |
| --- | --- |
| Phase 1: Ethics approval and field preparation  1) Full ethics protocol submission  2) Preparatory visits to health facilities  3) Recruitment and training of research assistants/counselors  4) Pre-test and printing of questionnaires  Phase 2: Recruitment of 552 participants  1) Brief interventions of alcohol problems  2) Project management meetings, teleconferences  3) Interview Quality control  4) Data entry/ analysis and publication    Phase 3: First follow-up interviews with participants (3 months)  1) Interviews  2) Project management meetings  3) Quality control  4) Data entry/ analysis/Publication  Phase 4: Second follow-up interviews with participants (12 months)  1) Interviews  2) Project management meetings  3) Quality control  4) Data entry/ analysis/Publication | July-Aug 2011  Aug 2011  Aug 2011  Aug 2011  Sept-Nov 2011  Sept-Nov 011  Sept-Nov2011  Dec 2011  Dec-Feb 2012  Dec-Feb 2012  Dec- Feb 2012  Feb-Apr 2012  Oct-Dec 2012  Oct- Dec 2012  Oct- Dec 2012  Jan-Feb 2013 |

# Budget

| **Activities** | **Budget (R)** |
| --- | --- |
| Teleconference with research team and consultant in Belgium | R 4,000 |
| Salaries 2 full time field workers # R 6000 pm x 6 months | R 72,000 |
| Top up pharmacy assistant adherence measurement | R 5,000 |
| Training the field workers | R 2,000 |
| Pre-testing questionnaires | R 1,000 |
| Printing questionnaires, inform consent, reports | R 6,000 |
| Data/ interview quality control | R 3,000 |
| Recruitment and first interview (drink) R 6 per case x 552 | R 3,312 |
| 1^st^ and 2^nd^ follow up ( R 12 x 552) | R 6,624 |
| Telephone for follow up | R 4,000 |
| Data entry ( R 10 X 552 X 4) | R 22,080 |
| Publication fee and editing (expected 5 papers) | R 30,000 |
| **Total** | **R 159,016** |

# References

World Health Organisation 2011.Global status report on alcohol and health 2011.ISBN 978 92 4 1564151. Available on <http://www.who.int/substance_abuse/publications/global_alcohol_report/msbgsruprofiles.pdf> (accessed on 2-5-2011).

2World Health Organisation. Global status report on alcohol and health 2011. Country profile on alcoholconsumption- South Africa.Available on http://www.who.int/substance_abuse/publications/en/south_africa.pdf (accessed on 2-5-2011).

3World Health Organisation.Epidemiological Fact Sheeton HIV and AIDS Core data on epidemiology and response. South Africa. 2008 update. Available at [http://apps.who.int/globalatlas/predefinedReports/EFS2008/full/EFS2008 _ZA.pdf](http://apps.who.int/globalatlas/predefinedReports/EFS2008/full/EFS2008%20_ZA.pdf) (accessed on 2-5-2011).

4 Bassett MT, McFarland WC, Ray S, Mbizvo MT, Machekano R, Van de Wijgert JHHM, Katzenstein DA. Risk factors for HIV infection at enrollment in an urban male factory cohort in Harare, Zimbabwe. *J Acquir Immune Defic Syndr Hum Retrovirol,* 1996;13(3):287-293.

5 Ayisi JG, Van Eijk AM, Ter Kuile FO, Kolczak MS, Otieno JA, misore AO, Kager PA, Steketee RW, Nahlen BL. Risk factors for HIV infection among asymptomatic pregnant women attending an antenatal clinic in western Kenya. *Int J STD AIDS,* 2000; 11(6):393-401.

6Fritz KE, Woelk GB, Bassett MT, McFarland WC, Routh JA, Tobaiwa O, Stall RD. The association between alcohol use, sexual risk behavior and HIV infection among men attending beerhalls in Harare, Zimbabwe. *AIDS and Behavior* 2002; 6(3):221-228.

7Hargreaves JR, Morison LA, Chege J, Rutenburg N, Kahindo M, Weiss HA, Hayes R, Buve A for the Study group on heterogeneity of HIV epidemics in African cities. Socioeconomic status and risk of HIV infection in an urban population in Kenya. *Trop Med Int Health* 2002, 7(9):793-802.

8 Mbulaiteyea SM, Ruberantwaria A, Nakiyingia JS, Carpenterb LM, Kamalia A, Whitwortha JAG. Alcohol and HIV: a study among sexually active adults in rural southwest Uganda. *Int J Epidemiol*, 2000; 29(5):911-915.

9Campbell C, Williams B, Gilgen D.Is social capital a useful conceptual tool for exploring community level influences on HIV infection? An exploratory case study from South Africa. *Aids Care* 2002, 14(1):41-54.

^0^ Clift S, Anemona A, Watson-Jones D, Kanga Z, Ndeki L, Changalucha J, Gavyole A, Ross DA. Variations of HIV and STI prevalences within communities neighbouring new goldmines in Tanzania: importance for intervention design. *Sex Transm Infect* 2003,79:307-312.

^1^ Fisher JC, Bang H, Kapiga SH. The association between HIVinfection and alcohol use: A systematic review and meta-analysisof African studies. *Sex Transm Di,* 2007; 34:856–863.

^2^Fisher JC, Cook PA, Kapiga SH. Alcohol use before sex and HIV risk: situational characteristics of protected and unprotected encounters among high-risk African women. *Sex Trans Dis,* 2010;37(9):571-518

^3^Woolf-King S, Maisto SA. Alcohol use and high-risk sexual behavior in Sub-Saharan Africa: a narrative review. *Arch Sex Behav*, 2011;40:17-42.

^4^Peltzer K, Mlambo G, Phaweni K. Factors determining prenatal HIV testing for prevention of mother to child transmission of HIV in Mpumalanga, South Africa. *AIDS Behave,* 2010;14:1115-1123.

^5^Msuya SE, Mbizvo E, Uriyo J, Stray-Pedersen B, Sam NE, Hussain A. Predictors of failure to return for HIV test results among pregnant women in Moshi, Tanzania. *J Acquir Immune Defic Syndr* 2006; 43:85-90.

^6^ Abaynew Y, Deribew A, Deribe K. Factors associated with late presentation to HIV/AIDS care in South Wollo Zone Ethiopia: a case-control study. *AIDS Res Ther*, 2011; 8:8.

^7^ Kip E, Ehlers VJ, van der Wal DM. Patients’ adherence to anti-retroviral therapy in Botswana. J Nurs Scholarsh. 2009, 41(2):149-157.

^8^Venkatesh KK, Srikrishnan AK, Mayer KH, Kumarasamy N, Raminani S, Thamburaj E, Prasad L, Triche EW, Solomon S, Safren SA. Predictors of nonadherence to highly active antiretroviral therapy among HIV-infected South Indians in clinical care: implications for developing adherence interventions in resource-limited settings. *AIDS Patient Care* STDS, 2010; 24(12):795-803.

^9^Jaquet A, Ekouevi DK, Bashi J, Aboubakrine M, Messou E, Malga M, Traore HA, Zannou MD, Guehi C, Ba-Gomis FO, Minga A, Allou G, Eholie SP, Bissagnene E, Sasco AJ, Dabis F. Alcohol use and non-adherence to antiretroviral therapy in HIV-infected patients in West Africa. *Addiction,* 2010; 105(8):1416-1421.

2^0^ van Geertruyden JP, Woelk G. Mukumbi H, Ryder R, Colebunders R. Alcohol and antiretroviral adherence? What about Africa? *J AIDS,* 2010; 54(4):e10.

^2^Etienne M, Hossain M, Redfield R, Stafford K, Amoroso A. Indicators of adherence to antiretroviral therapy treatment among HIV/AIDS patients in 5 African countries. *J Int Assoc Physicians AIDS Care,* 2010; 9:98-103.

^22^Baum MK, Rafie C, Lai S, Sales S, Page JB, Campa A. Alcohol use accelerates HIV disease progression. *AIDS Res Hum Retroviruses,*  2010; 26(5):511-518.

2^3^ Samet JH, Cheng DM, Libman H, Nunes DP, Alperen JK, Saitz R. Alcohol consumption and HIV disease progression. *J Acquir Immune Defic Syndr*, 2007; 46(2):194-199.

24 Conigliaro J, Gordon AJ, McGinnis KA, Rabeneck L, Justice AC for the Veterans Aging Cohort 3-site study. How harmful is hazardous alcohol use and abuse in HIV infection: do health care providers know who is at risk? *J AIDS* 2003; 33(4):521-525.

2^5^ Gmel G, Shield KD, Rehm J. Developing a method to derive alcohol-attributable fractions for HIV/AIDS mortality based on alcohol’s impact on adherence to antiretroviral medication. *Popul Health Metr,* 2011; 14(9(1):5.

2^6^ Papas RK, Sidle JE, Martino S, Baliddawa JB, Songole R, Omolo OE, Gakinya BN, Mwaniki MM, Adina JO, Nafula T, Owino-Ong’or WD, Bryant KJ, Carroll KM, Goulet JL, Justice AC, Maisto SA. Systematic cultural adaptation of cognitive-behavioral therapy to reduce alcohol use among HIV-infected outpatients in Western Kenya. *AIDS Behav*, 2010; 14:669-678.

2^7^ Hendershot CS, Stoner SA, Pantalone DW, Simoni JM. Alcohol use and antiretroviral adherence: review and meta-analysis. *J Acquir Immune Defic Syndr*. 2009; 52:180-202.

28 O’Connell K, Saxena S, Skevington SM & WHOQOLHIV group. WHOQOL-HIV for quality of life assessment among people living with HIV and AIDS: Results from a field test. *AIDS Care*, 2004; 16(7):882-889.

2^9^ World Health Organization (WHO). WHOQOLHIV instrument user’s manual: scoring and coding for the WHOQOL-HIV Instruments. Geneva 2002. Available on: <http://www.who.int/mental_health/media/en/613.pdf>.

3^0^ Andresen EM, Malmgren JA, Carter WB, Patrick DL. Screening for depression in well older adults: evaluation of a short form of the CES-D (Center for Epidemiologic Studies Depression Scale). *Am J Prev Med*, 1994; 10(2):77-84.

3^1^ Kilbourne AM, Justice AC, Rollman BL, McGinnis KA, Rabeneck L, Weissman S, Smola S, Schultz R, Whittle J, Rodriguez-Barradas M. Clinical importance of HIV and depressive symptoms among veterans with HIV infection. *J Gen Intern Med*, 2002; 17(7):512-520.

3^2^ Mulrow CD, Williams JW Jr, Gerety MB, Ramirez G, Montiel OM, Jerber C. Case-finding instruments for depression in primary care settings. *Ann Intern Med*, 1995; 122(12):913-921.

3^3^ Kalichman SC, Simbayi LC, Cloete A, Mthembuc PP, Mkhontac RN, Ginindza T. Measuring AIDS stigmas in people living with HIV/AIDS: The internalized AIDS related Stigma Scale. *AIDS Care,* 2009; 21(1):87-93.

3^4^ Babor TF, Higgins-Biddle JC. Brief intervention for hazardous and harmful drinking. A manual for use in primary care settings*.* World Health Organization, Geneva, Department of Mental Health and Substance Dependence. 2001. WHO/MSD/MSB/01.6b.

3^5^ Maneesriwongul WL, Tulathong S, Fennie KP, Williams AB. Adherence to antiretroviral medication among HIV-positive patients in Thailand. *J Acquir Immune Defic Syndr*. 2006; 43(Suppl. 1): S119-S122.

36 Sarna A, Luchters S, Geibel S, Munyao P, Kaai S, Khadija S, Kishor M, Hawken M, Van Dam J, Temmerman M. Promoting adherence through a directly administered antiretroviral therapy (DAART) strategy in Mombasa, Kenya. Horizons Research Update. 2005 Nairobi: Population Council.

^37^ Kalichman SC, Amaral CM, Cherry C, Flanagan J, Pope H, Eaton L, White D, Kalichman MO, Chain D, Deterio M, Caliendo A, Schinazi RF. Monitoring medication adherence by unannounced pill counts conducted by telephone: reliability and criterion-related validity. HIV Clin Trials. 2008;9(5):298-308.

3^8^ Department of Health.Republic of South Africa.Clinical guidelines for the management of HIV & AIDS in adults and adolescents.Available on: <http://www.doh.gov.za/docs/factsheets/guidelines/adult_art.pdf> (accessed on 2-5-2011)

3^9^ World Health Organization 2010.Antiretroviral therapy for HIV infection in adults and adolescents. Recommendations for a public health approach. 2010 Revision. Available on: [http://whqlibdoc.who.int/ publications/2010/9789241599764_eng.pdf](http://whqlibdoc.who.int/%20publications/2010/9789241599764_eng.pdf) (accessed on 2-5-2011).

# Annexes

#### Annex 1: Patient interview schedule and Demographic Characteristics

Interviewer: _______________________________

Place of Interview:__________________________

Start Time of Interview:_______________________

End Time of Interview:_______________________

P.I.D.______________________________________

**CONFIDENTIAL**

| **Patient Interview Schedule** | | |
| --- | --- | --- |
| Interview type | [ ] 1. Baseline Visit  [ ] 2. 1st Follow-up (3 months)  [ ] 3. 2nd Follow-up (12 months) | |
| Current physical address of participant:  ____________________________________________________________________________________________ | Name of village/suburb:_____________________________________  _____________________________________________________  Name of neighbour: ________________________________________  ______________________________________________  Nearest shop, church, school (name):__________________________  _____________________________________________________ | |
| Possible new physical address within a one year, if yes, detail as above: | _______________________________________________________________________________________________________________________________________________________________ | |
| Health facility name: | _____________________________________________________ | |
|  |  |  |
| Result codes  [ ] 1. Completed  [ ] 2. Respondent not available  [ ] 3. Respondent refused  [ ] 4. Partially completed  [ ] 5. Other (specify)_________________________________________________________________ | |  |

| **Part 1. Socio-demographic data-English** | | | | |
| --- | --- | --- | --- | --- |
| 1.1 How would you describe yourself in terms of population group? | [ ] 1. Black African  [ ] 2. Coloured | [ ] 3. Indian or Asian  [ ] 4. White | | [ ] 5. Other (specify) |
| 1.2 What language do you speak most often in your household? | [ ] 1. Afrikaans  [ ] 2. English  [ ] 3. IsiNdebele  [ ] 4. Isiswati  [ ] 5. IsiXhosa | [ ] 6. IsiZulu  [ ] 7. Sesotho sa borwa  [ ] 8. Sepedi  [ ] 9. Setswana  [ ] 10. Tshivenda | | [ ] 11. Xitsonga  [ ] 12. Other African  [ ] 13. Other European  [ ] 14. Indian language  [ ] 15. Other (specify) |
| 1.3 Age in completed years | ................................. | | | |
| 1.4 Gender | [ ] 1. Male | [ ] 2. Female | | |
| 1.5 What is your current marital status? | [ ] 1. Never married  [ ] 2. Currently married  [ ] 3. Separated | [ ] 4. Divorced  [ ] 5. Widowed  [ ] 6. Living together like married partners (cohabiting) | | |
| 1.6 What is your highest educational qualification? TICK ONE ANSWER ONLY | [ ] 1. No schooling  [ ] 2. Primary education (Grade 1-6)  [ ] 3. Secondar educatuion (Grade 7-12) | | [ ] 4. Diploma(s) / Occupational certificate(s)  [ ] 5. First degree(s)/ Higher diploma(s)  [ ] 6. Honours / Master’s degree(s) and higher | |
| 1.7 What is the main source of your household income? | [ ] 1. Formal salary/earnings on which you pay income tax  [ ] 2. Contributions by adult family members or relatives  [ ] 3. Contributions by younger family members or relatives (<18 years)  [ ] 4. Government pensions/grants (e.g. old age pension, disability grant)  [ ] 5. Grants/donations by private welfare organizations  [ ] 6. Other (specify)___________________________________________________________  [ ] 7. No income (other than social grant)____________________________________________ | | | |
| 1.8 What is the type of neighbourhood where you live? | [ ] 1. Rural village  [ ] 2. Informal settlements  [ ] 3. Urban/Town/metropolitan area  [ ] 4. Township  [ ] 5. Farm | | | |

#### Annex 2: Health-related quality of life (QoL)

###### Quality of life

| **Health-related quality of life** | | | | | | | | | | | | |
| --- | --- | --- | --- | --- | --- | --- | --- | --- | --- | --- | --- | --- |
| 1. How would you rate your quality of life? | [ ] 1. Very poor | [ ] 2. Poor | | | [ ] 3. Neither poor nor good | | [ ] 4. Good | | | [ ] 5. Very good | | |
| 2 How satisfied are you with your health? | [ ] 1. Very dissatisfied | [ ] 2. Dissatisfied | | | [ ] 3. Neither satisfied nor dissatisfied | | [ ] 4. Satisfied | | | [ ] 5. Very satisfied | | |
| The following questions ask about **how much** you have experienced certain things in the last two weeks. | | | | | | | | | | | | |
|  | | | **Not at all** | **A little** | | **A moderate amount** | | | **Very much** | | | **An extreme amount** |
| 3 To what extent does physical pain prevent you from doing what you need to do? | | | [ ] 1. | [ ] 2. | | [ ] 3. | | | [ ] 4. | | | [ ] 5. |
| 4 How much are you bothered by physical problems related to HIV/AIDS? | | | [ ] 1. | [ ] 2. | | [ ] 3. | | | [ ] 4. | | | [ ] 5. |
| 5 How much do you need any medical treatment to function in your daily life? | | | [ ] 1. | [ ] 2. | | [ ] 3. | | | [ ] 4. | | | [ ] 5. |
| 6 How much do you enjoy life? | | | [ ] 1. | [ ] 2. | | [ ] 3. | | | [ ] 4. | | | [ ] 5. |
| 7 To what extent do you feel your life to be meaningful? | | | [ ] 1. | [ ] 2. | | [ ] 3. | | | [ ] 4. | | | [ ] 5. |
| 8 To what extent are you bothered by people blaming you for having HIV/AIDS? | | | [ ] 1. | [ ] 2. | | [ ] 3. | | | [ ] 4. | | | [ ] 5. |
| 9 How much do you fear for the future because of HIV/AIDS? | | | [ ] 1. | [ ] 2. | | [ ] 3. | | | [ ] 4. | | | [ ] 5. |
| 10 How much do you worry about death because of HIV/AIDS? | | | [ ] 1. | [ ] 2. | | [ ] 3. | | | [ ] 4. | | | [ ] 5. |
| 11 How well are you able to concentrate? | | | [ ] 1. | [ ] 2. | | [ ] 3. | | | [ ] 4. | | | [ ] 5. |
| 12 How safe do you feel in your daily life? | | | [ ] 1. | [ ] 2. | | [ ] 3. | | | [ ] 4. | | | [ ] 5. |
| 13 How healthy is your physical environment? | | | [ ] 1. | [ ] 2. | | [ ] 3. | | | [ ] 4. | | | [ ] 5. |
|  | | | **Not at all** | **A little** | | **Moderately** | | | **Mostly** | | | **Completely** |
| 14 Do you have enough energy for everyday life? | | | [ ] 1. | [ ] 2. | | [ ] 3. | | | [ ] 4. | | | [ ] 5. |
| 15 Are you able to accept your bodily appearance? | | | [ ] 1. | [ ] 2. | | [ ] 3. | | | [ ] 4. | | | [ ] 5. |
| 16 Have you got enough money to meet your needs? | | | [ ] 1. | [ ] 2. | | [ ] 3. | | | [ ] 4. | | | [ ] 5. |
| 17 To what extent do you feel accepted by the people you know, whether or not they are aware of your HIV/AIDS? | | | [ ] 1. | [ ] 2. | | [ ] 3. | | | [ ] 4. | | | [ ] 5. |
| 18 How available to you is the information that you need in your day-to-day life? | | | [ ] 1. | [ ] 2. | | [ ] 3. | | | [ ] 4. | | | [ ] 5. |
| 19 To what extent do you have the opportunity for leisure activities? | | | [ ] 1. | [ ] 2. | | [ ] 3. | | | [ ] 4. | | | [ ] 5. |
| 20 How well are you able to get around? | | | [ ] 1. | [ ] 2. | | [ ] 3. | | | [ ] 4. | | | [ ] 5. |
|  | | | **Very dissatisfied** | **Dissatisfied** | | **Neither satisfied nor dissatisfied** | | | **Satisfied** | | | **Very satisfied** |
| 21 How satisfied are you with your sleep? | | | [ ] 1. | [ ] 2. | | [ ] 3. | | | [ ] 4. | | | [ ] 5. |
| 22 How satisfied are you with your ability to perform your daily activities? | | | [ ] 1. | [ ] 2. | | [ ] 3. | | | [ ] 4. | | | [ ] 5. |
| 23 How satisfied are you with your capacity to work? | | | [ ] 1. | [ ] 2. | | [ ] 3. | | | [ ] 4. | | | [ ] 5. |
| 24 How satisfied are you with yourself? | | | [ ] 1. | [ ] 2. | | [ ] 3. | | | [ ] 4. | | | [ ] 5. |
| 25 How satisfied are you with your personal relationships? | | | [ ] 1. | [ ] 2. | | [ ] 3. | | | [ ] 4. | | | [ ] 5. |
| 26 How satisfied are you with your sex life? | | | [ ] 1. | [ ] 2. | | [ ] 3. | | | [ ] 4. | | | [ ] 5. |
| 27 How satisfied are you with the support you get from your friends? | | | [ ] 1. | [ ] 2. | | [ ] 3. | | | [ ] 4. | | | [ ] 5. |
| 28 How satisfied are you with the condition of your living space? | | | [ ] 1. | [ ] 2. | | [ ] 3. | | | [ ] 4. | | | [ ] 5. |
| 29 How satisfied are you with your access to health services? | | | [ ] 1. | [ ] 2. | | [ ] 3. | | | [ ] 4. | | | [ ] 5. |
| 30 How satisfied are you with your transport? | | | [ ] 1. | [ ] 2. | | [ ] 3. | | | [ ] 4. | | | [ ] 5. |
| **The following question refers to how often you have felt or experienced certain things in the last two weeks.** | | | | | | | | | | | | |
| 31 How often do you have negative feelings such as depressed mood, despair, anxiety? | [ ] 1. Never | [ ] 2. Seldom | | | [ ] 3. Quite often | | | [ ] 4.Very often | | | [ ] 5. Never | |

###### Depression:

| **CES-D** | | | | |
| --- | --- | --- | --- | --- |
| **For each of these statements, please indicate how often you felt this way during the past week** | | | | |
|  | Rarely (<1day) | Some/little (1-2 days) | Much  (3-4 days) | Most  (5-7 days) |
| 1. I was bothered by things that usually don’t bother me | 1 | 2 | 3 | 4 |
| 2. I had trouble keeping my mind on what I was doing | 1 | 2 | 3 | 4 |
| 3. I felt that everything I did was an effort | 1 | 2 | 3 | 4 |
| 4. I felt depressed | 1 | 2 | 3 | 4 |
| 5. I felt hopeful about the future | 1 | 2 | 3 | 4 |
| 6. I felt fearful | 1 | 2 | 3 | 4 |
| 7. My sleep was restless | 1 | 2 | 3 | 4 |
| 8. I was happy | 1 | 2 | 3 | 4 |
| 9. I felt lonely | 1 | 2 | 3 | 4 |
| 10. I could not get going | 1 | 2 | 3 | 4 |

###### Sexual Behaviour

| **Sexual behaviour** | | | | | | |
| --- | --- | --- | --- | --- | --- | --- |
| **Please think carefully about the past 3-months and mark your answers**. | | | | | | |
| 1. Have you had sexual intercourse in the past 3 months | Yes [ ] | No [ ] | If no, go to next question | | | |
| 1. How many men have you had sex with in the past 3 months? | 0 | 1 | 2 | 3 | 4 | 5 or more men |
| 1. How many women have you had sex with in the past 3 months? | 0 | 1 | 2 | 3 | 4 | 5 or more men |

Now please think carefully about the past 3 months.

**In the past 3 months –How many times did you……**

| 1. Have sex **without** condoms (protection)? | 0 | 1-2 | 3-5 | 6-10 | 11-20 | 21-30 | 31-40 | 41 or more times |
| --- | --- | --- | --- | --- | --- | --- | --- | --- |
| 1. Have sex **with** use of condoms (protection)? | 0 | 1-2 | 3-5 | 6-10 | 11-20 | 21-30 | 31-40 | 41 or more times |
| 1. Drank alcohol before sex? | 0 | 1-2 | 3-5 | 6-10 | 11-20 | 21-30 | 31-40 | 41 or more times |
| 1. Your sex partner drank alcohol before sex? | 0 | 1-2 | 3-5 | 6-10 | 11-20 | 21-30 | 31-40 | 41 or more times |
| 1. Use drugs (Dagga, mandrax, or others) before sex? | 0 | 1-2 | 3-5 | 6-10 | 11-20 | 21-30 | 31-40 | 41 or more times |
| 1. Your sex partner used drugs (Dagga, mandrax, or others) before sex? | 0 | 1-2 | 3-5 | 6-10 | 11-20 | 21-30 | 31-40 | 41 or more times |

| 1. Use a condom at last sex, with partner whom unknown or negative HIV status | [ ] 1. Yes [ ] 2. No |
| --- | --- |
| 1. **In the past 3 months –I have** Disclosed my HIV status to my last sex partner | [ ] 1. Yes [ ] 2. No |

###### Internalized AIDS stigma

| **Internalised stigma** | | | | |
| --- | --- | --- | --- | --- |
|  | **Strongly Agree** | **Agree** | **Disagree** | **Strongly Disagree** |
| 1 It is difficult to tell other people about my HIV infection | [ ] 1. | [ ] 2. | [ ] 3. | [ ] 4. |
| 2 I am ashamed that I am HIV positive | [ ] 1. | [ ] 2. | [ ] 3. | [ ] 4. |
| 3 I sometimes feel worthless because I am HIV positive | [ ] 1. | [ ] 2. | [ ] 3. | [ ] 4. |
| 4 I feel guilty that I am HIV positive | [ ] 1. | [ ] 2. | [ ] 3. | [ ] 4. |
| 5 I hide my HIV status from others | [ ] 1. | [ ] 2. | [ ] 3. | [ ] 4. |
| 6 Being HIV positive makes me feel dirty | [ ] 1. | [ ] 2. | [ ] 3. | [ ] 4. |
| 7 It is my own fault that I am HIV positive | [ ] 1. | [ ] 2. | [ ] 3. | [ ] 4. |

#### Annex 3: Alcohol consumption

| **The Alcohol Use Disorders Identification Test (AUDIT): Interview Version-English** | | | | | | |
| --- | --- | --- | --- | --- | --- | --- |
| Dear Patient,  As part of my service I am examining lifestyle issues likely to affect the health of my patients. This information is important because it will assist me in giving the best treatment and highest possible standard of care. To help me do this, now I would like to ask you some questions about your use of alcohol beverages during the past 3 months. Your answers to these questions will be treated in strict confidence. No details of your name will be written on this questionnaire.  **Age:** ____ years **Gender:** [1] Male [2] Female **Date*:*** ____/ ____/ _______  A standard drink is: | | | | | | |
| *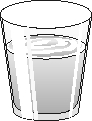* | *A single tot of spirits (whisky, gin & vodka)*  *(e.g., 25ml at 43%)* | *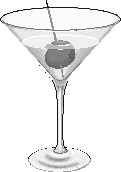* | *A small glass of liqueur or aperitif*  *(e.g., 25ml at 30%)* | | | |
| *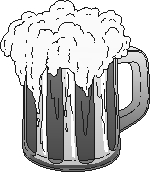* | *1 can of ordinary beer*  *(e.g., 340ml at 5%)* | *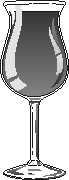* | *1 glass of wine*  *(e.g., 120ml at 12%)* | *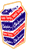* | *Cartoon of ordinary commercial sorghum beer*  *(e.g., 500ml at 3%)* | |
| 1. How often do you have a drink containing alcohol in the past 3 months?  Never [*skip to Qs 9-10*]……………………………….  Monthly or less.........................................................  2 to 4 times a month…………………………………..  2 to 3 times a weeks…………………………………..  4 or more times a week...……………………….......... | | (0)  (1)  (2)  (3)  (4)  [___] | 6. How often during the last 3 months have you needed a first drink in the morning to get yourself going after a heavy drinking session?  Never…………………………………………...  Less than monthly……………………………..  Monthly…………………………………………  Weekly……………………………………….....  Daily or almost daily …………………………. | | | (0)  (1)  (2)  (3)  (4)  [___] |
| 2. How many drinks containing alcohol do you have on a typical day when you are drinking?  1 or 2……………………………………..……...……  3 or 4…………………………………………..……...  5 or 6……………………………………………….....  7, 8 or 9………………………………………………...  10 or more……………………………………………... | | (0)  (1)  (2)  (3)  (4)  [___] | 7. How often during the last 3 months have you had a feeling of guilt or remorse after drinking?  Never…………………………………..……….  Less than monthly………………………..……  Monthly…………………………………….......  Weekly……………………………………........  Daily or almost daily………………………..… | | | (0)  (1)  (2)  (3)  (4)  [___] |
| 3. How often do you have (for men) five or more and (for women) four or more drinks on one occasion?  Never  Less than Monthly  Monthly  Weekly  Daily or almost daily  ***Skip to question 9 and 10 if Total Score for Qs 2 and 3 is =0*** | | (0)  (1)  (2)  (3)  (4)  [___] | 8. How often during the last 3 months have you been unable to remember what happened the night before because you had been drunk?  Never  Less than monthly  Monthly  Weekly  Daily or almost daily | | | (0)  (1)  (2)  (3)  (4)  [___] |
| 4. How often during the last 3 months have you found that you were not able to stop drinking once you had started?  Never……………………………………………………  Less than monthly……………………………………..  Monthly………………………………………………….  Weekly…………………………………………………..  Daily or almost daily…………………………………... | | (0)  (1)  (2)  (3)  (4)  [___] | 9. Have you or someone else been injured as a result of your drinking?  No……………………………………………….  Yes, but not in the last 3 months…………….  Yes, during the last 3 months……………….. | | | (0)  (2)  (4)  [___] |
| 5. How often during the last year have you failed to do what was normally expected from you because of drinking?  Never……………………………………………………  Less than monthly……………………………………..  Monthly………………………………………………….  Weekly…………………………………………………..  Daily or almost daily………………………………… | | (0)  (1)  (2)  (3)  (4)  [___] | 10. Has a relative or friend or a doctor or another health worker been concerned about your drinking or suggested you to cut down?  No……………………………………………….  Yes, but not in the last 6 months…………….  Yes, during the last 6 months……………….. | | | (0)  (2)  (4)  [___] |
| Record total of specific items here [____] | | | | | | |

**USE OF Tobacco products**

| TOB.1 Do you **currently** use one or more of the following tobacco products (cigarettes, snuff, chewing tobacco, cigars, etc.)? | **Yes** | **No** |
| --- | --- | --- |
|  | 1 | 2 |

**If ‘No’, go to next section**

TOB.2 In the past month, how often have you used one or more of the following tobacco products (cigarettes, snuff, chewing tobacco, cigars, etc.)?

| Once or twice | 1 |
| --- | --- |
| Weekly | 2 |
| Almost daily | 3 |
| Daily | 4 |

**Questionaire in Setswana Version**

#### Patient interview schedule and Demographic Characteristics

| **1. Socio-demographic data-Tswana** | | | | |
| --- | --- | --- | --- | --- |
| 1.1 O ka itlhalosa gore o wa morafe ofe? | [ ] 1. Black African  [ ] 2. Coloured | [ ] 3. Indian or Asian  [ ] 4. White | | [ ] 5. Other (specify) |
| 1.2 Ke leleme lefe leo le le dirisang ka fa gae? | [ ] 1. Afrikaans  [ ] 2. English  [ ] 3. IsiNdebele  [ ] 4. Isiswati  [ ] 5. IsiXhosa | [ ] 6. IsiZulu  [ ] 7. Sesotho sa borwa  [ ] 8. Sepedi  [ ] 9. Setswana  [ ] 10. Tshivenda | | [ ] 11. Xitsonga  [ ] 12. Other African  [ ] 13. Other European  [ ] 14. Indian language  [ ] 15. Other (specify) |
| 1.3 Dingwaga tse o di fitlheletseng |  | | | |
| 1.4 O mong? | [ ] 1. Male | [ ] 2. Female | | |
| 1.5 Maemo a gago a lenyalo? | [ ] 1. Never married  [ ] 2. Currently married  [ ] 3. Separated | [ ] 4. Divorced  [ ] 5. Widowed  [ ] 6. Living together like married partners (cohabiting) | | |
| 1.6 Dithuto tse o di fitlheletseng? Tlhopha karabo e le nngwe fela | [ ] 1. No schooling  [ ] 2. Primary education (Grade 1-6)  [ ] 3. Secondar educatuion (Grade 7-12) | | [ ] 4. Diploma(s) / Occupational certificate(s)  [ ] 5. First degree(s)/ Higher diploma(s)  [ ] 6. Honours / Master’s degree(s) and higher | |
| 1.7 Lotseno lwaka fa gae ke le le ntseng jang? | [ ] 1. Lotseno lo lo tlwaelegileng leo o duelang lekgetho.  [ ] 2. Contributions by adult family members or relatives  [ ] 3. Contributions by younger family members or relatives (<18 years)  [ ] 4. Government pensions/grants (e.g. old age pension, disability grant)  [ ] 5. Grants/donations by private welfare organizations  [ ] 6. Other (specify)_______________________________________________  [ ] 7. No income (other than social grant) | | | |
| 1.8 Ke mmano a ntseng jang a o nnang mo go one? | [ ] 1. Rural village  [ ] 2. Informal settlements  [ ] 3. Urban/Town/metropolitan area  [ ] 4. Township  [ ] 5. Farm | | | |

#### Health-related quality of life (QoL)-Setswana

###### Quality of life-SeTswana

| **Health-related quality of life** | | | | | | | | | | | | |
| --- | --- | --- | --- | --- | --- | --- | --- | --- | --- | --- | --- | --- |
| 1. O ka ipaya mo selekanong sefe ka maemo a bophelo ba gago? | [ ] 1. Tshotlego e e feteletseng | [ ] 2. Tshotlego | | | [ ] 3. Magareng | | [ ] 4. Bontle | | | [ ] 5. Bontle bo bo feteletseng | | |
| 2. O kgotsofetse go le kae ka bophelo ba gago? | [ ] 1. Go se kgotsofale go go feteletseng | [ ] 2. Go se kgotsofale | | | [ ] 3. Magareng | | [ ] 4. Go kgotsofala | | | [ ] 5. Go kgotsofala go go freteletseng | | |
| Dipotso tse di latelang di go botsa ka gore o itse go le kae ka dilo tse o di dirileng mo dibekeng tse pedi tse di fitileng. | | | | | | | | | | | | |
|  | | | Le go ka | Go le gonnye | | Mo go lekaneng | | | Go le gontsi | | | Mo go feteletseng |
| 3. Botlhoko ba mmele bo go thibela go le kae mo go direng dilo tse o tlhokang go di dira? | | | [ ] 1. | [ ] 2. | | [ ] 3. | | | [ ] 4. | | | [ ] 5. |
| 4. O tshwenyega go le kae ka mathata a mmele a a tsamaisanang le HIV/AIDS? | | | [ ] 1. | [ ] 2. | | [ ] 3. | | | [ ] 4. | | | [ ] 5. |
| 5. O tlhoka go le kae go dirisa melemo go tswelela mo letsatsing? | | | [ ] 1. | [ ] 2. | | [ ] 3. | | | [ ] 4. | | | [ ] 5. |
| 6. Botshelo bo go itumedisa go le kae? | | | [ ] 1. | [ ] 2. | | [ ] 3. | | | [ ] 4. | | | [ ] 5. |
| 7. O utlwa botshelo ba gago bo le botlhokwa go le kae? | | | [ ] 1. | [ ] 2. | | [ ] 3. | | | [ ] 4. | | | [ ] 5. |
| 8. O tshwenyega go le kae ka batho ba go kgoba ka gonne o na le bolwetse ba HIV/AIDS? | | | [ ] 1. | [ ] 2. | | [ ] 3. | | | [ ] 4. | | | [ ] 5. |
| 9. O tshoga go le kae ka bokamoso ba gago ka lebaka la HIV/AIDS? | | | [ ] 1. | [ ] 2. | | [ ] 3. | | | [ ] 4. | | | [ ] 5. |
| 10. O tshwenyega go le kae ka lefu ka lebaka la HIV/AIDS? | | | [ ] 1. | [ ] 2. | | [ ] 3. | | | [ ] 4. | | | [ ] 5. |
| 11. O kgona go le kae go reetsa ka tlhoafalo? | | | [ ] 1. | [ ] 2. | | [ ] 3. | | | [ ] 4. | | | [ ] 5. |
| 12. O ikutlwa o sireletsegile go le kae mo matsatsing a gago a bophelo? | | | [ ] 1. | [ ] 2. | | [ ] 3. | | | [ ] 4. | | | [ ] 5. |
| 13. Legae le o dulang mo go lona le tlhwekile go le kae? | | | [ ] 1. | [ ] 2. | | [ ] 3. | | | [ ] 4. | | | [ ] 5. |
|  | | | Le go ka | Go le gonnye | | Mo go lekaneng | | | Go le gontsi | | | Mo go feeletseng |
| 14. A o na le maikatlapelo a a lekaneng go tshela letsatsi le letsatsi? | | | [ ] 1. | [ ] 2. | | [ ] 3. | | | [ ] 4. | | | [ ] 5. |
| 15. O kgona go amogela seemo se o lebegang ka sona? | | | [ ] 1. | [ ] 2. | | [ ] 3. | | | [ ] 4. | | | [ ] 5. |
| 16. O na le madi a a lekaneng go fitlhelela ditlhokego tsa gago? | | | [ ] 1. | [ ] 2. | | [ ] 3. | | | [ ] 4. | | | [ ] 5. |
| 17. O ikutlwa o amogetswe go le kaego le kae ke batho ba ba itseng seemo sa gago sa HIV/AIDS? | | | [ ] 1. | [ ] 2. | | [ ] 3. | | | [ ] 4. | | | [ ] 5. |
| 18. Kitso e e tlhokagalang e teng go le kae mo matsatsing a gago a bophelo? | | | [ ] 1. | [ ] 2. | | [ ] 3. | | | [ ] 4. | | | [ ] 5. |
| 19. O kgona go le kae go fitlhelela sebaka sa go iketla? | | | [ ] 1. | [ ] 2. | | [ ] 3. | | | [ ] 4. | | | [ ] 5. |
| 20. O kgona jang go tswelela? | | | [ ] 1. | [ ] 2. | | [ ] 3. | | | [ ] 4. | | | [ ] 5. |
|  | | | Go se kgotsofale mo go feteletseng | Go se kgotsofale | | Magareng | | | Go kgotsofala | | | Go kgotsofala go go feteletseng |
| 21. O kgotsofala go le kae ka boroko ba gago? | | | [ ] 1. | [ ] 2. | | [ ] 3. | | | [ ] 4. | | | [ ] 5. |
| 22. O kgotsofala go le kae ka mokgwa o o kgonang go dira ditiro tsa gago tsa letsatsi? | | | [ ] 1. | [ ] 2. | | [ ] 3. | | | [ ] 4. | | | [ ] 5. |
| 23. O kgotsofala go le kae ka bokgone ba gago ba go dira ditiro tsa gago tsa letsatsi? ? | | | [ ] 1. | [ ] 2. | | [ ] 3. | | | [ ] 4. | | | [ ] 5. |
| 24. O kgotsofala go le kae ka ga wena? | | | [ ] 1. | [ ] 2. | | [ ] 3. | | | [ ] 4. | | | [ ] 5. |
| 25. O kgotsofala go le kae ka bokgotsi ba gago? | | | [ ] 1. | [ ] 2. | | [ ] 3. | | | [ ] 4. | | | [ ] 5. |
| 26. O kgotsofala go le kae ka bophelo ba gago ba thobalano? | | | [ ] 1. | [ ] 2. | | [ ] 3. | | | [ ] 4. | | | [ ] 5. |
| 27. O kgotsofala go le kae ka kemonokeng e metswalle ya gago e go neang yone? | | | [ ] 1. | [ ] 2. | | [ ] 3. | | | [ ] 4. | | | [ ] 5. |
| 28. O kgotsofala go le kae ka maemo a o dulang mo go ona? | | | [ ] 1. | [ ] 2. | | [ ] 3. | | | [ ] 4. | | | [ ] 5. |
| 29. O kgotsofala go le kae ka go fitlhelela tsa pholo? | | | [ ] 1. | [ ] 2. | | [ ] 3. | | | [ ] 4. | | | [ ] 5. |
| 30. O kgotsofala go le kae ka senamelwa se o se dirisang? | | | [ ] 1. | [ ] 2. | | [ ] 3. | | | [ ] 4. | | | [ ] 5. |
| Dipotso tse di latelang di botsa go le kae ka dilo tse o di dirileng mo dibeke tse pedi tse di fitileng. | | | | | | | | | | | | |
| 31. Ke ga kae o ikutlwa o na le letshogo e bile moya wa gago o le ko tlase? | [ ] 1. Le go ka | [ ] 2. Go le gonnye | | | [ ] 3. Go le gontsi | | | [ ]4.Go le gontsi thata | | | [ ] 5. Le go ka | |

###### Depression-Setswana

CES-D: Mo tse di latelang, tlhopa e e tsamaisanang le ka mokgwa o o neng o ikutlwa ka ona mo bekeng e e fitileng

| **CES-D** | | | | |
| --- | --- | --- | --- | --- |
|  | Go se kalo(<1day) | Go le gonnye(1-2 days) | Go le gontsi (3-4 days) | Go le gontsi thata (5-7 days) |
| a. Ke tshwentse ke dilo tse gantsi di sa tshwenyeng | 1 | 2 | 3 | 4 |
| b. Ke bile le bothata ba go tsepamisa tlhaloganyo ya me mo se ke neng ke se dira | 1 | 2 | 3 | 4 |
| c. Ke utlwile dilo tse tsotlhe tse ne ke dira e le matsapa | 1 | 2 | 3 | 4 |
| d. Maikutlo a me a ne a ya ko tlase | 1 | 2 | 3 | 4 |
| e. Ke ikutlwile ke ba le tshepo ka bokamoso | 1 | 2 | 3 | 4 |
| f. Ke ikutlwile ke boifa | 1 | 2 | 3 | 4 |
| g. Ke ne ke tlhobaelwa | 1 | 2 | 3 | 4 |
| h. Ke ne ke thabile | 1 | 2 | 3 | 4 |
| i. Ke ikutlwile ke le bodutu | 1 | 2 | 3 | 4 |
| j. Ke ne ka palelwa ke go tswelela | 1 | 2 | 3 | 4 |

###### Sexual Behaviour-Setswana

| **Sexual behaviour** | | | | | | |
| --- | --- | --- | --- | --- | --- | --- |
| Nagana sentle ka ga dikgwedi tse 3 tse di fitileng mme o tlhophe karabo ya gago. | | | | | | |
| 1. O ne wa tsena mo thobalanong mo kgweding tse tharo tse di fitileng? | Ee [ ] | Nya [ ] | Ga o re nya, e ya ko potso ya bolesome | | | |
| 2. O robalane le borre ba le bakae mo kgweding tse tharo tse di fitileng? | 0 | 1 | 2 | 3 | 4 | 5 kgotsa ba go feta |
| 3. O robalane le bomme ba le bakae mo kgweding tse tharo tse di fitileng? | 0 | 1 | 2 | 3 | 4 | 5 kgotsa ba go feta |

Jaanong nagana sentle ka ga dikgwedi tse 3 tse di fitileng.

Mo kgweding tse 3 tse di fitileng-Ke makgetho a le makae……

| 4. O tsene mo thobalanong ka ntle le go dirisa kgotlhopo? | 0 | 1-2 | 3-5 | 6-10 | 11-20 | 21-30 | 31-40 | 41 kgotsa ba go feta |
| --- | --- | --- | --- | --- | --- | --- | --- | --- |
| 5. O tsene mo thobalanong o dirisa kgotlhopo? | 0 | 1-2 | 3-5 | 6-10 | 11-20 | 21-30 | 31-40 | 41 kgotsa ba go feta |
| 6. O tsene mo thobalanong o nwele bojalwa? | 0 | 1-2 | 3-5 | 6-10 | 11-20 | 21-30 | 31-40 | 41 kgotsa ba go feta |
| 7. Molekane wa gago a nwele bojalwa pele le robalana? | 0 | 1-2 | 3-5 | 6-10 | 11-20 | 21-30 | 31-40 | 41 kgotsa ba go feta |
| 8. O dirisitse diritibatsi pele ga thobalano? | 0 | 1-2 | 3-5 | 6-10 | 11-20 | 21-30 | 31-40 | 41 kgotsa ba go feta |
| 9. Molekane wa gago a dirisitse diritibatsi pele ga thobalano? | 0 | 1-2 | 3-5 | 6-10 | 11-20 | 21-30 | 31-40 | 41 kgotsa ba go feta |

| 10. O ne wa se dirise kgolhopo le molekane yo o sa itseng maemo a gagwe a HIV | [ ] 1. Ee [ ] 2. Nya |
| --- | --- |
| **11. Mo kgweding tse 3 tse di fitileng –Ke itsisitse** Molekane wa bofelo o ke neng ka tsena mo thobalanong le ena ka maemo a me a HIV | [ ] 1. Ee [ ] 2. Nya |

###### Internalized AIDS stigma-Setswana

| **Internalised stigma** | | | | |
| --- | --- | --- | --- | --- |
|  | Go dumela mo go feteletseng | Go dumela | Go se dumele | Go se dumele mo go feteletseng |
| 1. Go boima go bolelela batho ba bangwe ka maemo a ka a HIV | [ ] 1. | [ ] 2. | [ ] 3. | [ ] 4. |
| 2. Ke swabisa ke gore ke HIV+ | [ ] 1. | [ ] 2. | [ ] 3. | [ ] 4. |
| 3. Ka nako tse dingwe ke ikutlwa ke se na boleng ka gonne ke le HIV+ | [ ] 1. | [ ] 2. | [ ] 3. | [ ] 4. |
| 4. Ke ikutlwa ke molato ka go ba HIV+ | [ ] 1. | [ ] 2. | [ ] 3. | [ ] 4. |
| 5. Ke fitlha maemo a me a HIV go ba bangwe | [ ] 1. | [ ] 2. | [ ] 3. | [ ] 4. |
| 6. Go ba HIV+ go dira gore ke ikutlwe ke le maswe | [ ] 1. | [ ] 2. | [ ] 3. | [ ] 4. |
| 7. Ke molato wa me ga ke le HIV+ | [ ] 1. | [ ] 2. | [ ] 3. | [ ] 4. |

####

#### Alcohol consumption-SeTswana

| **The Alcohol Use Disorders Identification Test (AUDIT):**  **Interview Version- Tswana** | | | | | | |
| --- | --- | --- | --- | --- | --- | --- |
| Molwetsi yo o rategang,  Jaana ka e nngwe ya ditsela tseo ke di dirisang go tlhatlhoba maemo a mathata a tsamaisanang le tsa pholo mo botshelong jwa gago. Kitso eo ke tla e fitlhelang e botlhokwa ka gore e tla nthusa go neelana ka thuso kana go oka le tlhokomelo e e kwa godimo. Gore ke kgone go dira seno, ke tla kopa gore o orabe dipotso tse di latelang ka tiriso ya nnotagi mo dikgweding tse thataro tse di fetileng. Dikarabo tsa dipotso tseo di tla beng di boditswe di tla nna mo sephiring. Mo pampiring ya dipotso ga go kitla go ntshiwa sepe se se amanang le leina la gago.  **Age:** ____ Dingwaga **Gender:** [1] Monna [2] Mosadi **Letlha*:*** ____/ ____/ _______  Nnotagi e e lekaneng e: | | | | | | |
| *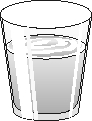* | *Thoto e le nngwe ya sepiriti (whisky, gin & vodka) (e.g., 25ml at 43%)* | *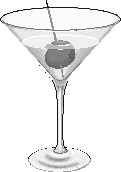* | *Galase e le nngwe ya lekaraese*  *(e.g., 25ml at 30%)* | | | |
| *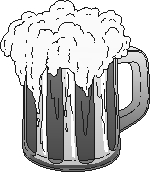* | *Khene e le nngwe ya bojalwa*  *(e.g., 340ml at 5%)* | *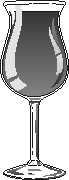* | *Galase e le nngwe ya beine*  *(e.g., 120ml at 12%)* | *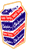* | *Katone e le nngwe ya bojalwa ba setho*  *(e.g., 500ml at 3%)* | |
| 1. Ke ka makgetlho a le makae o ne o nwa seno se se nang le bojalwa mo dikgweding tse thataro tse di fetileng?  Le goka [*tlolela go Qs 9-10*]……………………….  Kgwedi kana kwa tlase……………………………....  Gabedi go ya go gane mo kgweding……………….  Gabedi go ya go gararo mo bekeng……................  Gane kgotsa go feta mo bekeng…………………. | | (0)  (1)  (2)  (3)  (4)  [__] | 6. Ke ga kae mo dikgweding tse thataro tse di fetileng mo o neng o tlhoka nnotagi fela mosong go tlosa letsapa la nno e ntsi e o nnileng le yone mo bosigong jo bo fetileng?  Le goka...........................................................  Mo tlase ga kgwedi.........................................  Kgwedi nngwe le nngwe.................................  Beke nngwe le nngwe....................................  Tsatsi lengwe le lengwe................................. | | | (0)  (1)  (2)  (3)  (4)  [__] |
| 2. Mo letsatsing ke dinnotagi di le kae tseo o di nwang?  1kgotsa 2…………………………………………….  3kgotsa 4…………………………………………….  5kgotsa 6…………………………………………….  7, 8, kgotsa 9…………………………………………  10 kgotsa go fetisa…………………………………. | | (0)  (1)  (2)  (3)  (4)  [__] | 7. Ke ga kae mo dikgweding tse thataro tse di fetileng nneng wa itshola fa morago ga go nwa nnotagi?  Le goka...........................................................  Mo tlase ga kgwedi.........................................  Kgwedi nngwe le nngwe.................................  Beke nngwe le nngwe....................................  Tsatsi lengwe le lengwe................................. | | | (0)  (1)  (2)  (3)  (4)  [__] |
| 3. Ke ka makgetlho a le kae (fa o le monna) tse tlhano kana go feta le (fa o le mosadi) dinnotagi di le nne kgotsa go feta mo kopanong e le nngwe?  Le goka...................................................................  Mo tlase ga kgwedi.................................................  Kgwedi nngwe le nngwe.........................................  Beke nngwe le nngwe.............................................  Tsatsi lengwe le lengwe..........................................  ***Tlolela ko potsong ya 9 and 10 if Total Score for Qs 2 and 3 is =0*** | | (0)  (1)  (2)  (3)  (4)  [__] | 8. Ke ka makgetlho a makae mo dikgweding tse thataro mo e leng gore o paletswe ke go gopola gore go diragetseng bosigo bo bo fetileng ka gonne o ne o nole?  Le goka..........................................................  Mo tlase ga kgwedi.........................................  Kgwedi nngwe le nngwe.................................  Beke nngwe le nngwe....................................  Tsatsi lengwe le lengwe................................. | | | (0)  (1)  (2)  (3)  (4)  [__] |
| 4. Ke ka makgetlho a le makae mo dikgweding di le thataro tse di fetileng mo o fitlhetseng e le gore fa o simolola go nwa nno-tagi o palelwa ke go emisa?  Le goka...................................................................  Mo tlase ga kgwedi.................................................  Kgwedi nngwe le nngwe.........................................  Beke nngwe le nngwe.............................................  Tsatsi lengwe le lengwe.......................................... | | (0)  (1)  (2)  (3)  (4)  [__] | 9. A go mongwe kgotsa wena o kile wa gobala ka ntlha ya nnotagi?  Nnya ya…………………………………………..  Ee, e seng mo dikgweding tse thatarotse di  fetileng…………………………………………  Ee, Mo dikgweding tsedithataro tse di fetileng | | | (0)  (2)  (4)  [__] |
| 5. Ke ka makgetlho a le makae mo ngwageng o fetileng mo o reteletsweng ke go dira seo o neng o tshwanetse ke go se dira ka ntlha ya nnotagi ya gago?  Le goka...................................................................  Mo tlase ga kgwedi.................................................  Kgwedi nngwe le nngwe.........................................  Beke nngwe le nngwe............................................  Tsatsi lengwe le lengwe......................................... | | (0)  (1)  (2)  (3)  (4)  [__] | 10. A mongwe wa losika kgotsa tsala, ngaka, kgotsa modiredi wa tsa pholo yo o kileng a tshwenyega ka go nwa nnotagi ga gago kgotsa a rotloetsa gore o fokotse bojalwa?  Nnya ya…………………………………………..  Ee, e seng mo dikgweding tse thatarotse di  fetileng…………………………………………  Ee, Mo dikgweding tsedithataro tse di fetileng | | | (0)  (2)  (4)  [__] |
| Rekoto ya ditiriso tse di maleba [____] | | | | | | |

**Tiriso ya metsoko e e farologaneng:**

| TOB.1 A ka se sebaka o dirisa mongwe kgotsa mengwe ya metsoko e e latelang (sakarete, seneifi, motsoko o o tlhafoniwang, disigara, le tse dingwe.)? | **Ee** | **Nyaa** |
| --- | --- | --- |
|  | 1 | 2 |

**Fa osa dumalelane’, e ya kwa letlheng le lengwe**

TOB.2 Mo kgweding e e fetileng, ke ga kae o dirisa e mongwe kgotsa go feta ga mefuta ya metsoko e e latelang (cigarettes, snuff, chewing tobacco, cigars, etc.)?

| Gangwe kgotsa gabedi | 1 |
| --- | --- |
| Beke nngwe le nngwe | 2 |
| Go isa go letsatsi ka letsatsi | 3 |
| Letsatsi lengwe le lengwe | 4 |

#### Annex 4: Patients Information Extract Sheet: HIV related information

| **HIV related information** | | | | | | | |
| --- | --- | --- | --- | --- | --- | --- | --- |
| INTERVIEWER: Please record the latest readings from the medical file of the following: | | | | | | | |
| **Date start ART: _______/_______/______________** | | | | | | | |
| **Lab and clinical** | | | | | | | |
| 1. CD4 Count:_____________________cells/mm^3^ | | | | | | | |
| 1. Viral Load:______________________copies/ml | | | | | | | |
| 1. Opportunistic infections:   __________________________________________________________________________________________________  ____________________________________________________________________________________________________________________________________________________________________________________________________________________ | | | | | | | |
| 1. **WHO clinical stage:** 1. [ ] 2. [ ] 3. [ ] 4. [ ] | | | | | |  | |
| 1. **HIV medication** | | |  | | | | |
| **NRTI**   1. Tenofovir (TDF).......... 2. Lamivudine (3TC) ...... 3. Zidovudine (AZT)........ 4. Stavudine (d4T).......... 5. Emtricitabine (FTC).... 6. Other.......................... | | [ ]  [ ]  [ ]  [ ]  [ ]  [ ] | **NNRTI**   1. Efavirenz (stocrin, EFV) …… 2. Nevirapine (NVP)…………… 3. Other………………………… | [ ]  [ ]  [ ] | **Protease inhibitor (PI)**   1. Lopinavir/ritonavir (LPV/r) (Aluvia)……….. 2. Other…………………. | | [ ] [ ] |
| 1. **Medications for HIV-related opportunistic infections:** | | | | | | | |
| a. | Cotrimoxazole (Nucotrim / Bactrim / Cozole / Purbac) | | | [ ] 1. Yes [ ] 2. No | | | |
| b. | TB treatment 1^st^ line (e.g Rifinah, Rifafour, Rifater, Rimstar, Ethatyl) | | | [ ] 1. Yes [ ] 2. No | | | |
| c. | TB treatment 2^nd^ line (e.g. Cycloserine) | | | [ ] 1. Yes [ ] 2. No | | | |
| d. | Acyclovir | | | [ ] 1. Yes [ ] 2. No | | | |
| e. | Fluconazole (Diflucan) | | | [ ] 1. Yes [ ] 2. No | | | |
| f. | Ciprofloxacin | | | [ ] 1. Yes [ ] 2. No | | | |
| g. | Miconazole (Daktarin) | | | [ ] 1. Yes [ ] 2. No | | | |
| h. | Phenoxymethylpenicillin (e.g. Len V.K.) | | | [ ] 1. Yes [ ] 2. No | | | |
| i. | Anti-diarrhoeal (e.g. Loperamide) | | | [ ] 1. Yes [ ] 2. No | | | |
| j. | Other (specify)______________________________________________ | | | [ ] 1. Yes [ ] 2. No | | | |
| 1. **Non-HIV medications:** | | | | | | | |
| a. | Vitamins (incl. Pyridoxine, Thiamine, Folic acid, Biocare Potassium) | | | [ ] 1. Yes [ ] 2. No | | | |
| b. | Immune supplement (e.g. Moducare) | | | [ ] 1. Yes [ ] 2. No | | | |
| c. | Blood pressure treatment (antihypertensive e.g. Methodopa) | | | [ ] 1. Yes [ ] 2. No | | | |
| d. | Anusol (for piles) | | | [ ] 1. Yes [ ] 2. No | | | |
| e. | Diabetes treatment (e.g. Metformin, Dia-care) | | | [ ] 1. Yes [ ] 2. No | | | |
| f. | Anti-inflammatory (e.g. Brufen, Ibuprofen, Inza) | | | [ ] 1. Yes [ ] 2. No | | | |
| g. | Pain-killer (analgesic e.g. Grandpa) | | | [ ] 1. Yes [ ] 2. No | | | |
| h. | Diuretic (e.g. Ridaq) | | | [ ] 1. Yes [ ] 2. No | | | |
| i. | Heart palpitations treatment (e.g. Cocillana) | | | [ ] 1. Yes [ ] 2. No | | | |
| j. | Emollient, skin cream (e.g. Epizone benzoic) | | | [ ] 1. Yes [ ] 2. No | | | |
| k. | Other :  (specify)______________________________________________ | | | [ ] 1. Yes [ ] 2. No | | | |

#### Annex 5. Assessment of adherence to ART (compiled by the Pharmacy assistant)

###### Visual Analog Scale (VAS)

| **VAS adherence measure** | |
| --- | --- |
| Most patients find it difficult to take all their HIV medications exactly as prescribed. | |
| 1 How many doses of your HIV medication did you miss in the last 7 days? | _____________________________ (# of doses) |
| 2 Please put a mark on the line below at the point that shows your best guess about how much of your prescribed HIV medication you have  taken in the last month.  Examples: 0% means you have taken no medication  50% means you have taken half your medication  100% means you have taken every single dose of your medication  Visual Analogue Scale 0 10 20 30 40 50 60 70 80 90 100 | |

Patient self report:

###### Pill count

#### Annex 6: Health education leaflet

**LETLAKALA LA THUTO YA TSA BOITEKANELO: Ke eng se se isang kelo ya nnotagi kwa godimo?**

Go nwa nno-agi go fetisa selekano go ka baka tse di latelang:

*Mathata a go ka laola maikutlo*

*Mathata a le kamano le batho ba o tshelang le bone*

*Mathata a mala a botlhoko*

*Letshololo*

*Go tlhoka boroko bosigo*

*Go wela fa fatshe kana dikotsi tse dingwe*

*Go tshwarwa ke mapodisi ka ntlha ya go nwa nno-tagi go feta selekano*

*Mathata a go tlhoka madi (tshelete)*

*Go kopana tlhogo morago ga go nwa nno-tagi*

*Bolwetse jwa maikutlo*

*Go latlhegelwa ke mogopolo*

*Mathata le go nwa melemo ee fodisang*

*Dikotsi tsa sejanaga*

*Go ikutlwa o le nosi ka ntlha ya nnotagi*

*Kelo ya madi e e kwa godimo*

*Go roroma ga diatla*

*Fa mmele o tlhoka dikotla tse di maleba*

*Bolwetse jwa go wa*

*Bolwetse jwa kankere*

*Bolwetse jwa go swa letlhakore kana seterouko*

*Go nwa nnotagi go go feteletseng go ka tlisa pholo e e kwa tlase kgotsa go fetoga ga mekgwa le dikamano tsa gago. O ka tswa o nnile le maiphitlhelo a tse dingwe tsa mathata ao re buileng ka ona fa godimo a tsamaisanang le go nwa nnotagi.*

**Gopola seno:**

Go nwa nno-tagi mo selekanong ke go go latelang:

**Bomme:**

- Se nwe go feta dinnotagi tse pedi fela ka letsatsi.
- Se nwe go feta matsatsi a le mabedi mo bekeng.

**Borre:**

- Se nwe go feta dinno-tagi tse tharo ka letsatsi.
- Se nwe go feta matsatsi a le mabedi mo bekeng.

**Batho botlhe ba ba leng mo godimo ga dingwaga di le 65**

- Se nwe go feta dinno- tagi tse pedi fela ka letsatsi.
- Se new go feta matsatsi a le mabedi mo bekeng.

**Gopola seno:**

Nnotagi e lekaneng ke e latelang:


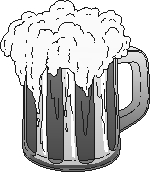


Thoto e le nngwe fela ya sepiriti (e.g., 25ml at 43%)

Khene e le nngwe fela ya bojalwa (e.g., 340ml at 5%)


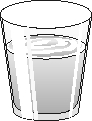


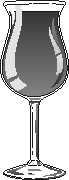


Galase e le nngwe ya beine.(e .g., 120ml at 12%)


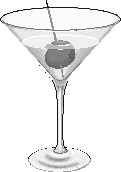


Galase e le nngwe ya lekaraese. (e.g., 25ml at 30%)

#### Annex 7: Consent form

INFORMATION SHEET: STEP 1

Screening and brief intervention for alcohol problems in HIV outpatients in the clinics associated with Dr George Mukhari Hospital, Ga-Rankuwa, South Africa: A single-blinded randomized controlled trial.

Hello, I am …….. from the University of Limpopo and our organisation is asking people from this clinic to answer some questions.

**PURPOSE**

This research study is called Screening and brief intervention for alcohol problems in HIV outpatients in the clinics associated with Dr George Mukhari Hospital, Ga-Rankuwa, South Africa: A single-blinded randomized controlled trial. This study will test new ways to reduce important health risks. The purpose of this part of screening for health risks is to find out more about your risk of harmful alcohol use by doing a health risk screening test by questionnaire. Depending upon the results of this test, you will be offered further management.

**POTENTIAL BENEFITS**

This health risk screening test will show whether you might be at elevated risk for a health problem. You may choose to not find this out by not taking part in this test.

**PROCEDURES**

You will be asked questions about your health status and health risk behaviour. This will take about 30 minutes. If the results from this screening test show that it might be all right for you to take part in the study, you will be invited for an intervention. Also we will collect some basic information from your patientfile and laboratory results regarding your HIV infection.

**RISKS AND DISCOMFORTS**

You might feel uncomfortable about some of the questions asked of you. You may choose not to answer any questions.

**INFORMATION SHEET**

Please understand that you are not being forced to take part in this study, and the choice to participate or notis yours alone. However, we would really appreciate it if you would be willing to assist us. If you choose not to take part in answering these questions, you will not be affected in any way. You will not lose any benefits that you would normally be entitled to receive from medical services agencies. If you choose to participate, you will not be paid for participating, and you will not receive any preferential treatment from medical services agencies. If you do participate, you may refuse to answer any question that you do not want to answer, and you may stop the interview at any time. If you do this there will also be no penalties and you will NOT be prejudiced in ANY way.

Breaches of confidentiality of someone’s information may be a potential risk in this study. However, extensive procedures have been put in place to ensure that this will not occur. Our staff has been trained to carefully follow detailed procedures designed to assure that no information about any person will be released to anyone outside the research team. All information provided by you will be treated as strictly confidential and will only be used by people in the research project. Your name will not be linked to any of your answers. Only the most senior researchers on the project will have access to both your name and your answers. The only place where names will appear is on this informed consent form, indicating agreement to take part in this study. These forms will be kept in a locked filing cabinet, which will be kept separate from the interview. Codes will be used to protect identity.

If you feel that you have been harmed in any way by participating in this study, or If you have any questions about your rights as a study participant, or questions or concerns about any aspect of the study, you may contacplease call the Research Principal Investigator, Prof. Supa Pengpid on 012 521 5036, or cell 0833276477.

This information sheet is for you to keep.

###### CONSENT FORM: STEP 1

Screening and brief intervention for alcohol problems in HIV outpatients in the clinics associated with Dr George Mukhari Hospital, Ga-Rankuwa, South Africa: A single-blinded randomized controlled trial.

**CONSENT**

I hereby agree to participate in research regarding health risk prevention.I understand that I am participating freely and without being forced in any way to do so. I also understand that I can stop this interview at any point should I not want to continueand that this decision will not in any way affect me negatively.

I understand that this is a research project whose purpose is not necessarily to benefit me personally.

I have received the telephone number of a person to contact should I need to speak about any issues which may arise in this interview.

I understand that this consent form will not be linked to the questionnaire, and that my answers will remain confidential.

I understand that if at all possible, feedback will be given to my community on the results of the completed research.

Participant Name (printed) Participant Code

Signature of Participant Date

Interviewer Signature Date

*If verbal consent is provided, the interviewer must sign below in the presence of the participant and a witness.*

(Signature of interviewer certifying that informed Date

consent has been given verbally by respondent)

(Signature of witness certifying that informed Date

consent has been given verbally by respondent)

INFORMATION SHEET: STEP 2

Screening and brief intervention for alcohol problems in HIV outpatients in the clinics associated with Dr George Mukhari Hospital, Ga-Rankuwa, South Africa: A single-blinded randomized controlled trial.

**PURPOSE AND BACKGROUND**

This research study is called Screening and brief intervention for alcohol problems in HIV outpatients in the clinics associated with Dr George Mukhari Hospital, Ga-Rankuwa, South Africa: A single-blinded randomized controlled trial. This study will test new ways to prevent health risks such as risky drinking.

With this in mind, the purpose of this health risk intervention is to find out whether risk reduction counselling will reduce alcohol risk in persons who have a medium alcohol risk score. We have found that you have a medium alcohol risk score. If you had screened a high risk score you would have been directed to specialist services.

**POTENTIAL BENEFITS**

Screening tests have been done that show you may take part in this study. These screening tests have shown that you are at medium alcohol use risk. Your taking part in this research will help us learn how to prevent or reduce alcohol risk. Your taking part in this study might prevent or reduce alcohol risk. You also have the choice of not joining this study.

**PROCEDURES**

Now that you are ready to begin the intervention, you will be put into a group by chance; like flipping a coin placed in one of two groups. You will not be able to choose your group. You will be put into a group by chance (like flipping a coin), either Group A or Group B.

1 Group A: Receive one session brief counselling (15-20 minutes) on alcohol use. You are also requested to come for follow-up assessments (which can also happen by phone) after 3 and 12 months, linked to your usual follow up visits to the clinic.

2 Group B: Receive a health information leaflet and you are also requested to come for follow-up assessments after 3 and 12 months, linked to your usual follow up visits to the clinic. After 12 months you will receive the brief counselling intervention.

Also we will collect information from your patientfile and laboratory results regarding your HIV infection.

**RISKS AND DISCOMFORT**

You might feel uncomfortable about some of the questions asked of you or some of the counselling content. You may choose not to answer any questions or stop the counselling session.

CONSENT FORM: STEP 2

Screening and brief intervention for alcohol problems in HIV outpatients in the clinics associated with Dr George Mukhari Hospital, Ga-Rankuwa, South Africa: A single-blinded randomized controlled trial.

Consent

I hereby agree to participate in research regarding health risk prevention and reduction.I understand that I am participating freely and without being forced in any way to do so. I also understand that I can stop this brief intervention at any point should I not want to continueand that this decision will not in any way affect me negatively.

I understand that this is a research project whose purpose is not necessarily to benefit me personally.

I have received the telephone number of a person to contact should I need to speak about any issues which may arise in this interview.

I understand that this consent form will not be linked to the questionnaire, and that my participation will remain confidential.

I understand that if at all possible, feedback will be given to my community on the results of the completed research.

Participant Name (printed) Participant Code

Signature of Participant Date

Interviewer Signature Date

*If verbal consent is provided, the interviewer must sign below in the presence of the participant and a witness.*

(Signature of interviewer certifying that informed Date

consent has been given verbally by respondent)

(Signature of witness certifying that informed Date

consent has been given verbally by respondent)

#### Annex 8: Consent form Tswana

**Mametlelelo 3: Foromo ya tumelelo**

INFORMATION SHEET: STEP 1

Screening and brief intervention for alcohol problems in HIV outpatients in the clinics associated with Dr George Mukhari Hospital, Ga-Rankuwa, South Africa: A single-blinded randomized controlled trial.

**Papetlana ya tshedimosetso: Kgato 1**

**Tsereganyo ya Africa Borwa ya matshosetsi a boitekanelo**

Dumela, Ke nna …….. go tswa unibesiting ya Limpopo mme mokgatlho wa rona o botsa batho go tswa mo tleliniking e dipotso.

**MAIKAELELO**

Patlisiso e e bidiwa lenaneo la tshekatsheko ya dikotsi tsa pholo mo Afrika Borwa. Patlisiso e e sekaseka ditsela tse ntshwa tsa go fokotsa tshwaetsego ya malwetse. Maikaelelo magolo a diteko tse ke go lebelela kotsi ya go dirisa nno-tagi mo go feteletseng mo pholong ya gago ka go botsa dipotso mo pampitshaneng e. Fa re se na go lebelela maemo a dipholo tsa diteko tseo re tla bong re di dirile, mme o tla fiwa tsela ya phodiso kana tshekatsheko ya bothata.

**DIKUNO TSE DI TSHEPISANG**

Teko e etla dirwang e tla bontsha fa o le mo kotsing ya go tshwaetsega ke bolwetse. O ka nna wa itlhophela go se tsee karolo mo patlisisong e.

**DITSAMAISO**

O tla bodiwa dipotso ka seemo sa pholo ya gago le ka mokgwa o o tshelang ka teng go ka tshwaetsega ke malwetse. Seo se tla tsaya metsotso e le lesome fela. Fa dipholo tsa diteko tse di bontsha gore o ka tsaya karolo mo patlisisong e, o tla bidiwa gore o nne motsayakarolo mo patlisisong go ya pele.

**DIKOTSI LE DITLAMORAGO**

Fa o ikutlwa o sa nnisege sentle ka dipotso tse di ka bodiwang. O ka nna wa tlhopha go se arabe dipotso tseo.

**KITSO E NNGWE**

O tshwanetse o utlwisise gore ga o patelediwe gore o tseye karolo mo patlisisong e, mme tshwetso ya go tsaya karolo kgotsa nnyaya e sala le wena. Le fa go le jalo, re ka itumela fa o ka tsaya karolo mo go thusaneng le rona go bona kitso. Fa e le gore o tlhopha go se tseye karolo mo patlisisong, o ka se amege ka gope. O ka se latlhegelwe ke dikuno tseo o ntseng o di bona fa o ile go batla thuso kwa ditirelong tsa kokelo. Fa e le gore o tlhopha go tsaya karolo, o ka se duelwe go tsaya karolo, o ka se fitlhele tshwaro kana thuso e e fetang mo metlheng go tswa mo ditirelong tsa pholo. Fa e le gore o tsaya karolo, o ka gana go araba tse dingwe tsa dipotso tseo o sa batleng go di araba, o ka kgaotsa patlisiso nako nngwe le nngwe fa o rata. Mme fa o dira se ga go ditshenyegelo dipe, o ka se atlholwe ka ntlha ya seo.

Go tlolwa ga tumalano ya khupamarama magareng ga bao ba dumelaneng e ka senya maemo a patlisiso gotlhelele. Le fa go le jalo, ditsamaiso tse di tseneletseng di beilwe go netefatsa gore seo se ka se direge. Bathapiwa ba ro rona ba rupisitswe tota gore ba latelele tsamaiso e e tseneletseng e e diretsweng gore kitso epe ka mongwe e se ntshetswe go ope kwa ntle ga patlisiso e. Kitso yotlhe e o re nayang yone e tla dirisiwa jaaka khupamarama mme e bile e tla dirisiwa fela ke batho ba ba leng mo patlisisong. Leina la gago le ka se amane gope le dikarabo tse di filweng. Ke batho fela ba ba leng kwa godimo mo porojekeng e, bao ba tla nnang le kitso ka maina le dikarabo. Moo go tla dirisiwang maina a batsayakarolo ke karolo ya foromo e e buwang ka tetlelelo fela, e e bontshang tumalano magareng ga maphate a mabedi.Diforomo tse di tla tswalelwa mo lefelong le le sireletsegileng, tseo di tla bewang kgakala le tsa dipotso. Dikhoutu kana matshwao a tla dirisiwa go sireletsa batsayakarolo.

Fa o ikutlwa o kgopisegile ka tsela nngwe le nngwe fela mo patlisisong e, kgotsa fa o na le dipotso mabapi le go lokologa ga gago mo patlisisong e, kgotsa dipotso dingwe le dingwe mo patlisisong e, o ka letsetsa the mmatlisisi yo mogolo, Prof. Supa Pengpid mo nomorong e, 012 521 5036, kgotsa mogala wa letheka 0833276477.

Letlakala le la patlisiso o ka le tshwara mo go wena fa o batla.

###### FOROMO YA TETLELELO: LENANEO LA NTLHA

Screening and brief intervention for alcohol problems in HIV outpatients in the clinics associated with Dr George Mukhari Hospital, Ga-Rankuwa, South Africa: A single-blinded randomized controlled trial.

**Tsereganyo ya Afrika Borwa ya matshosetsi a boitekanelo**

**TETLELELO**

Ke dumela go nayana ka kitsiso ya go thibela go nna le malwetse a afarologaneng.Ke ya tlhaloganya gore ke tsaya karolo mo patlisisong kwantle le go patelediwa ke ope . Ke a tlhaloganya gore nka emisa patlisiso e ka nako e nngwe le e nngwe fa ke batla kgotsa fa ke sa tlhole ke batla go tswelela pele ka yone le gore tshwetso eo fa ke e tsaya e ka se nkame ka tsela e sa siamang.

Ke tlhaloganya gore patlisiso e maikaelelo a yona ga se go tlisa dikuno mo go nna.

Ke bone dinomoro tsa mogala tseo ke tla kgonang go bua ka tseo di sa nkgotsofatseng mo patlisisong e.

Ke tlhaloganya gore foromo ya tetlelelo ga e amane ka gope le dipotso tseo ke tla di botswang mo patlisisong, le gore dikarabo tseo ke tla di nayang di tla sireletsega.

Ke a tlhaloganya gore fa go kgonega, ditlamorago tsa patlisiso di tla newa setshaba fa patlisiso e fela.

Participant Name (printed) Participant Code

Leina la Motseayakarolo Khouto ya Motseayakar

Signature of Participant Date

Tshaeno ya Motseakarolo Lrtlha

Interviewer Signature Date

Tshaeno ya Morerisanyi Letlha

*If verbal consent is provided, the interviewer must sign below in the presence of the participant and a witness.*

Fa e le gore go diragadiwa tumelelano ya molomo, morerisanyi o tla saena fa tlase fa pele ga motsayakarolo le paki.

(Signature of interviewer certifying that informed Date

consent has been given verbally by respondent) Lettlha

Tshaeno ya morerisanyi a ikana gore tumelelano e e itsegeng e ne e neilwe ke moikarabedi ka molomo(boena)

###### LETLAKALA LA KITSOKAKARETSO: LENANEO LA BOBEDI

Screening and brief intervention for alcohol problems in HIV outpatients in the clinics associated with Dr George Mukhari Hospital, Ga-Rankuwa, South Africa: A single-blinded randomized controlled trial.

**Tsereganyo ya Afrika Borwa ya matshosetsi a boitekanelo**

**MAIKAELELO LE LEMORAGO**

Patlisiso e e bidiwa lenaneo la tshekatsheko ya matshosetsi a pholo mo Afrika Borwa. Patlisiso e e sekaseka ditsela tse ntshwa tsa go thibela le go laola nno-tagi.

Ka seo mo tlhaloganyong, maikaelelo a tshekatsheko e ke go lebelela gore kgothatso le thuto ka ga dikotsi tsa nno-tagi e ka fokotsa kelo e kwa godimo y a nnotagi.Se re se boning ke gore go na le mola wa taolo ya go bona fa nnotagi e tswa mo taolong. Fa o bontsha nno-tagi e le kwa godimo mo go wena o romelwa kwa baitsanapeng gore o sekasekiwa go ya pele.

**DIKUNO TSE DI TSHEPISANG**

Screening tests have been done that show you may take part in this study. These screening tests have shown that you are at medium alcohol use risk. Your part in this research will help us learn how to prevent or reduce alcohol risk. Your taking part in this study might prevent or reduce alcohol risk. You also have the choice of not joining this study.

**DITSAMAISO**

Jaanong ka gore o ipaakanyeditse go nna karolo ya patlisiso, o tla bewa mo setlhopheng sengwe; o tla tsenngwa mo sengwe ya ditlhopha tse pedi. Ga o kitla o itlhophela setlhopha. O tla tsenngwa mo sengwe sa ditlhopa e ka nna setlhopha A kgotsa setlhopha B .

1 Setlhopha sa A: O tla bona thupelelo kana tshedimosetso ka go dirisa nnotagi mo metsotsong e le (15-20). O tla kopiwa gore o tle tshekatshekong e e tseneletseng (e e ka nna ya dirwa ka mogala) morago ga dikgwedi tse thataro le tse somamabedi.

2 Setlhopha sa B: O tla fitlhela letlakala la kitsokakaretso mme e bile o kopiwa go tla tshekatshekong e e tseneletseng (eo e ka dirwang ka mogala) morago ga dikgwedi tse thataro le tse somamabedi. Morago ga dikgwedi tse somamabedi o tla bona thotloetso e e tseneletseng e khutshwanyane.

**DIKOTSI LE DITLAMORAGO**

Fa o ikutlwa o sa nnisege sentle mo moyeng ka dipotso tse o di bodiwang kana thotloetso e sa go nniseng sentle. O ka itlhophela go se arabe dipotso tse di bodiwang kgotsa thupelelo e o e newang.

FOROMO YA TETLELELO: LENANEO LA BOBEDI

Screening and brief intervention for alcohol problems in HIV outpatients in the clinics associated with Dr George Mukhari Hospital, Ga-Rankuwa, South Africa: A single-blinded randomized controlled trial.

**TETLELELO**

Ke dumela go tsaya karolo mo patlisisong le tshedimosetso ya go thibela le go fokotsa kelo ya bolwetse.Ke tlhaloganya gore ke tsaya karolo ka go rata ga me kwa ntle le go pateletswa ke ope go dira jalo. E bile ke tlhaloganya gore nka emisa dipatlisiso nako nngwe le nngwe fa ke sa batle go tswelela pelele gore tshwetso eo ke tla bong ke e tsaya e ka se nkame ka tsela e sa siamang.

Ke a tlhaloganya gore se ke patlisiso eo maikaelelo a yone a sa lebanang le nna mme a akaretsa.

Ke fitlhetse dinomoro tsa mogala tsa motho yo o maleba yo nka buang le ene ka mathata a a ka tlhagelelang mo patlisisong.

Ke a tlhaloganya gore foromo ya tetlelelo ga e amane ka gope le foromo ya dipotso tsa patlisiso, le gore go tsaya karolo ga me go tla nna go sireletsegile.

Ke a tlhaloganya gore fa go kgonega, dipholo le ditlamorago tsa patlisiso di tla newa setshaba fa patlisiso e fela.

Participant Name (printed) Participant Code

Signature of Participant Date

Interviewer Signature Date

*If verbal consent is provided, the interviewer must sign below in the presence of the participant and a witness.*

(Signature of interviewer certifying that informed Date

Consent has been given verbally by respondent)

(Signature of witness certifying that informed Date

Consent has been given verbally by respondent)

**Alcohol Standard Units**

| *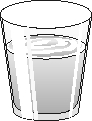* | *A single tot of spirits (whisky, gin & vodka) (e.g., 25ml at 43%)* |
| --- | --- |
| *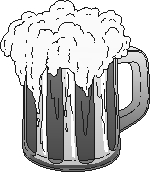* | *1 can of ordinary beer*  *(e.g., 340ml at 5%)* |
| *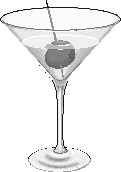* | A small glass of liqueur or aperitif *(e.g., 25ml at 30%)* |
| *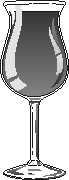* | *1 glass of wine*  *(e.g., 120ml at 12%)* |
| *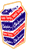* | *Cartoon of ordinary commercial sorghum beer*  *(e.g., 500ml at 3%)* |

**Appendix 1: Health Education Leaflet English**

HEALTH EDUCATION LEAFLET: What constitutes risky drinking?

Excessive drinking can result in:

Difficulty coping with stress

Relationship problems

Stomach problems

Diarrhoea

Sleeplessness

Falls or other accidents

Arrest for drinking under the influence of alcohol

Financial problems

Feeling confused after drinking

Depression

Memory loss

Problems with medications

Automobile accidents

Feeling alone or left out because of drinking

High blood pressure

Trembling hands

Malnutrition

Seizures

Cancer

Stroke

Drinking too much alcohol can hurt your health, your behaviours, and your relationships. You may have experienced some of the problems shown at the left, which may be related to your use of alcohol.

Remember:

Sensible drinking are:

Women:

- No more than 2 drinks per day
- Do not drink at least 2 days a week

Men:

- No more than 3 drinks per day
- Do not drink at least 2 days a week

**All persons over 65:**

- No more than 2 drinks per day
- Do not drink at least 2 days a week

**Remember:**

| 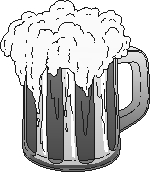 | A standard drink is:  One can of ordinary beer  (e.g.., 340ml at 5%) |
| --- | --- |
| 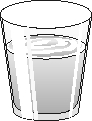 | A single tot of spirits  (e.g.., 52ml at 43%) |
| 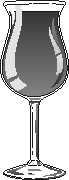 | One glass of wine  (e.g.., 120ml at 12%) |
| 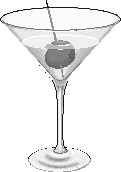 | A small glass of liqueur or aperitif  (e.g.., 25ml at 30%) |
| *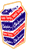* | Carton of ordinary commercial sorghum beer  (e.g., 500ml at 3%) |

**LETLAKALA LA THUTO YA TSA BOITEKANELO:**

**Ke eng se se isang kelo ya nnotagi kwa godimo?**

| Go nwa nno-agi go fetisa selekano go ka baka tse di latelang:  -Mathata a go ka laola maikutlo  -Mathata a le kamano le batho ba o tshelang le bone  -Mathata a mala a botlhoko  -Letshololo  -Go tlhoka boroko bosigo  -Go wela fa fatshe kana dikotsi tse dingwe  -Go tshwarwa ke mapodisi ka ntlha ya go nwa nno-tagi go feta selekano  -Mathata a go tlhoka madi (tshelete)  -Go kopana tlhogo morago ga go nwa nno-tagi  -Bolwetse jwa maikutlo  -Go latlhegelwa ke mogopolo  -Mathata le go nwa melemo ee fodisang  -Dikotsi tsa sejanaga  -Go ikutlwa o le nosi ka ntlha ya nnotagi  -Kelo ya madi e e kwa godimo  -Go roroma ga diatla  -Fa mmele o tlhoka dikotla tse di maleba  -Bolwetse jwa go wa  -Bolwetse jwa kankere  -Bolwetse jwa go swa letlhakore kana seterouko | Go nwa nnotagi go go feteletseng go ka tlisa pholo e e kwa tlase kgotsa go fetoga ga mekgwa le dikamano tsa gago. O ka tswa o nnile le maiphitlhelo a tse dingwe tsa mathata ao re buileng ka ona fa godimo a tsamaisanang le go nwa nnotagi.  **Gopola seno:** Go nwa nno-tagi mo selekanong ke go go latelang:  **Bomme:**   - Se nwe go feta dinnotagi tse pedi fela ka letsatsi. - Se nwe go feta matsatsi a le mabedi mo bekeng.   **Borre:**   - Se nwe go feta dinno-tagi tse tharo ka letsatsi. - Se nwe go feta matsatsi a le mabedi mo bekeng.   **Batho botlhe ba ba leng mo godimo ga dingwaga di le 65**   - Se nwe go feta dinno- tagi tse pedi fela ka letsatsi. - Se new go feta matsatsi a le mabedi mo bekeng.   **Gopola seno:** Nnotagi e lekaneng ke e latelang:  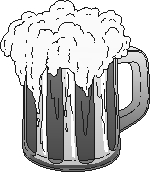  Khene e le nngwe fela ya bojalwa (e.g., 340ml at 5%)  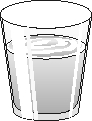  Thoto e le nngwe fela ya sepiriti (e.g., 25ml at 43%)  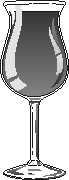  Galase e le nngwe ya beine. (e.g., 120ml at 12%)  Galase e le nngwe ya lekaraese (e.g., 25ml at 30%)  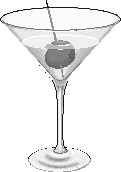  Carton of ordinary commercial sorghum beer  (e.g., 500ml at 3%)  *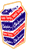* |
| --- | --- |

**LETLAKALA LA THUTO YA TSA BOITEKANELO:**

**Ke eng se se isang kelo ya nnotagi kwa godimo?**

| Go nwa nno-agi go fetisa selekano go ka baka tse di latelang:  -Mathata a go ka laola maikutlo  -Mathata a le kamano le batho ba o tshelang le bone  -Mathata a mala a botlhoko  -Letshololo  -Go tlhoka boroko bosigo  -Go wela fa fatshe kana dikotsi tse dingwe  -Go tshwarwa ke mapodisi ka ntlha ya go nwa nno-tagi go feta selekano  -Mathata a go tlhoka madi (tshelete)  -Go kopana tlhogo morago ga go nwa nno-tagi  -Bolwetse jwa maikutlo  -Go latlhegelwa ke mogopolo  -Mathata le go nwa melemo ee fodisang  -Dikotsi tsa sejanaga  -Go ikutlwa o le nosi ka ntlha ya nnotagi  -Kelo ya madi e e kwa godimo  -Go roroma ga diatla  -Fa mmele o tlhoka dikotla tse di maleba  -Bolwetse jwa go wa  -Bolwetse jwa kankere  -Bolwetse jwa go swa letlhakore kana seterouko | Go nwa nnotagi go go feteletseng go ka tlisa pholo e e kwa tlase kgotsa go fetoga ga mekgwa le dikamano tsa gago. O ka tswa o nnile le maiphitlhelo a tse dingwe tsa mathata ao re buileng ka ona fa godimo a tsamaisanang le go nwa nnotagi.  **Gopola seno:** Go nwa nno-tagi mo selekanong ke go go latelang:  **Bomme:**   - Se nwe go feta dinnotagi tse pedi fela ka letsatsi. - Se nwe go feta matsatsi a le mabedi mo bekeng.   **Borre:**   - Se nwe go feta dinno-tagi tse tharo ka letsatsi. - Se nwe go feta matsatsi a le mabedi mo bekeng.   **Batho botlhe ba ba leng mo godimo ga dingwaga di le 65**   - Se nwe go feta dinno- tagi tse pedi fela ka letsatsi. - Se new go feta matsatsi a le mabedi mo bekeng.   **Gopola seno:** Nnotagi e lekaneng ke e latelang:  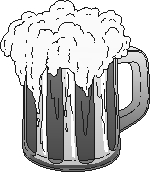  Khene e le nngwe fela ya bojalwa (e.g., 340ml at 5%)  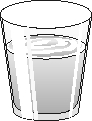  Thoto e le nngwe fela ya sepiriti (e.g., 25ml at 43%)  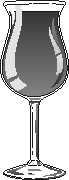  Galase e le nngwe ya beine. (e.g., 120ml at 12%)  Galase e le nngwe ya lekaraese (e.g., 25ml at 30%)  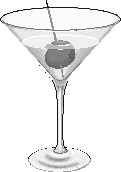  Carton of ordinary commercial sorghum beer  (e.g., 500ml at 3%)  *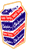* |
| --- | --- |

Date visit

Y

Y

Y

Y

M

M<

D

D

Name clinic: Phedisong 4 Soshanguve 3 Maria Rantho K.T. Motubatse

Name Team member_________________________________________________________________

m

m

h

h

Time in clinic :

m

m

h

hh

Time out clinic :

| Name patient | ID number | Tel number 1 | Tel number 2 | Inclusion study Y/N | Follow up date 3 months |
| --- | --- | --- | --- | --- | --- |
|  |  |  |  |  |  |
|  |  |  |  |  |  |
|  |  |  |  |  |  |
|  |  |  |  |  |  |
|  |  |  |  |  |  |
|  |  |  |  |  |  |
|  |  |  |  |  |  |
|  |  |  |  |  |  |
|  |  |  |  |  |  |
|  |  |  |  |  |  |
|  |  |  |  |  |  |
|  |  |  |  |  |  |
|  |  |  |  |  |  |

1. [↑](#endnote-ref-1)
